# Volitional modification of brain activity in adolescents with Autism Spectrum Disorder: A Bayesian analysis of Slow Cortical Potential neurofeedback

**DOI:** 10.1016/j.nicl.2021.102557

**Published:** 2021-01-09

**Authors:** L. Konicar, S. Radev, K. Prillinger, M. Klöbl, R. Diehm, N. Birbaumer, R. Lanzenberger, P.L. Plener, L. Poustka

**Affiliations:** a Department of Child and Adolescence Psychiatry, Medical University of Vienna, Vienna, Austria; b Institute of Psychology, University of Heidelberg, Germany; c Neuroimaging Labs, Department of Psychiatry & Psychotherapy, Medical University of Vienna, Austria; d Wyss Center for Bio and Neuroengineering, Geneva, Switzerland; e Department of Child and Adolescence Psychiatry, Medical University of Göttingen, Göttingen, Germany

**Keywords:** Slow Cortical Potential training, EEG Neurofeedback, Autism Spectrum Disorder, Adolescents, Bayesian multilevel model, Volitional brain activity modification

## Abstract

Autism spectrum disorder is (ASD) characterized by a persisting triad of impairments of social interaction, language as well as inflexible, stereotyped and ritualistic behaviors. Increasingly, scientific evidence suggests a neurobiological basis of these emotional, social and cognitive deficits in individuals with ASD.

The aim of this randomized controlled brain self-regulation intervention study was to investigate whether the core symptomatology of ASD could be reduced via an electroencephalography (EEG) based brain self-regulation training of Slow Cortical Potentials (SCP). 41 male adolescents with ASD were recruited and allocated to a) an experimental group undergoing 24 sessions of EEG-based brain training (n_1_ = 21), or to b) an active control group undergoing conventional treatment (n_2_ = 20), that is, clinical counseling during a 3-months intervention period. We employed real-time neurofeedback training recorded from a fronto-central electrode intended to enable participants to volitionally regulate their brain activity.

Core autistic symptomatology was measured at six time points during the intervention and analyzed with Bayesian multilevel approach to characterize changes in core symptomatology. Additional Bayesian models were formulated to describe the neural dynamics of the training process as indexed by SCP (time-domain) and power density (PSD, frequency-domain) measures.

The analysis revealed a substantial improvement in the core symptomatology of ASD in the experimental group (reduction of 21.38 points on the Social Responsiveness Scale, *SD* = 5.29), which was slightly superior to that observed in the control group (evidence Ratio = 5.79). Changes in SCP manifested themselves as different trajectories depending on the different feedback conditions and tasks. Further, the model of PSD revealed a continuous decrease in delta power, parallel to an increase in alpha power. Most notably, a non-linear (quadratic) model turned out to be better at predicting the data than a linear model across all analyses. Taken together, our analyses suggest that behavioral and neural processes of change related to neurofeedback training are complex and non-linear. Moreover, they have implications for the design of future trials and training protocols.

## Highlights

• Adolescents with Autism Spectrum Disorder were able to volitionally modify their brain activity.
• Improvements of core Autism Spectrum Disorder symptomatology are presented.
• Delta power band decreased, while Alpha power band increased during the course of neurofeedback training.
• Bayesian analysis revealed a non-linear pattern of neurofeedback-related changes.
• The complex picture of neural and behavioral processes of change is discussed.

## 1 Introduction

Autism spectrum disorder (ASD) is a neurodevelopmental disorder, comprising difficulties in developing and maintaining social relationships, failure of well-functioning verbal communication through reduced sharing of interests, motivation and activities, and decreased socio-emotional reciprocity, alongside with limited, inflexible and repetitive pattern of behavior (Organization WH, 2004).

Recent research suggests a neurobiological basis of the socio-emotional, cognitive and behavioral shortcomings of ASD. The anatomical signature seems to be marked by age-related structural changes, as recent findings in high-risk siblings suggest the presence of disrupted brain development in children with ASD before first behavioral symptoms emerge (Hazlett et al., 2017). Functional magnetic resonance imaging (fMRI) studies indicate abnormal brain activation in several cortical and limbic areas. Besides findings of ASD-specific dysfunctions in the anterior cingulate cortex (ACC, crucial for the integration of executive functions, motivational and emotional behaviors), aberrant activation has been reported in the medial and orbital regions of the frontal lobe, insula, uncus, fusiform gyrus, para- and hippocampal gyrus, cerebellum, as well as in the amygdala (for an overview of MRI findings see Ecker et al. (Ecker et al., 2015). Electrophysiological studies in ASD complement the structural and functional neuroimaging findings. Attenuated cortical excitability (e.g., indicated by a reduced Contingent Negative Variation (Hoofs et al., 2018); CNV), together with excessive slow-wave activity often in frontal brain areas (and interpreted as cortical hypoactivation) and reduced power in the alpha frequency band has been reported in several studies in ASD (e.g. Coben et al., 2008, Wang et al., 2013).

Taken together, the aberrant brain development, the abnormal activation found in fMRI studies, and the electrophysiological abnormalities seem to reflect altered cerebral excitability, functional integrity and neuroelectric synchronization between brain regions involved in socio-emotional and executive functioning.

Although ASD is a predominantly neurobiologically-based condition, behavioral and educational interventions are currently the primary treatments for addressing the core deficits in affected individuals. Moreover, these interventions often require long periods of time and intensive commitment (for a review, see Landa, 2018). Furthermore, core symptoms of ASD cannot be sufficiently treated with medication and pharmacology is mostly used to reduce comorbid psychiatric symptoms (Poustka and Kamp-Becker, 2016). Thus, there is hardly any doubt that a complex disorder such as ASD requires a multifaceted treatment approach. Considering adolescence as a transition period marked by social, cognitive and emotional adaptations (Ernst et al., 2006), parallel to research reporting dysfunctions in emotion regulation significant for the development and maintenance of mental disorders including autism (Ahmed et al., 2015), the need for interventions targeting especially the age period between 13 and 18 years in ASD is obvious. Therefore, neurobiologically-based approaches should, alongside behavior-based methods, be considered in the treatment of adolescents with ASD.

### Neurofeedback therapy

In the past decades, there have been several applications of neurofeedback in ASD in a clinical context, mostly confined to case reports or case studies using different frequency bands as a feedback signal⁠ (Cowan, 1994, Sichel et al., 1995). Still, only few systematic controlled studies investigating clinical outcomes of neurofeedback in ASD exist. These studies have focused on particular frequency bands, such as beta band (~16–31 Hz) and/or parallel theta band (4–8 Hz) (targeting beta increase/theta decrease) e.g. (Kouijzer et al., 2009) or based their feedback signal on individual quantitative EEG findings (e.g. Carrick et al., 2018).⁠ In the last years, yet another frequency-based neurofeedback approach was proposed, aimed at increasing EEG activity in the range of ~ 8–13 Hz, targeting the mirror neuron system (Datko et al., 2018, Pineda et al., 2014) (for frequency-band neurofeedback review and overview see Holtmann et al., 2011).

Another strand of neurofeedback research has explored the utility of neurofeedback training based on Slow Cortical Potentials (SCP) in disorders with neural dysregulation. SCP reflect changes in the depolarization level of the upper cortical layers and mirror local thresholds of excitability in cortical cell assemblies (He and Raichle, 2009, Birbaumer et al., 1990). SCP are very slow shifts in the EEG near to 0 Hz and can be subdivided into electrically negative shifts, which indicate excitatory mobilization, and electrically positive shifts, which indicate a reduction or inhibition of neuronal excitation (Birbaumer et al., 1990). Symptom-specific improvements after SCP self-regulation training have been demonstrated for disorders associated with hyperactivation, such as drug-resistant epilepsy (Strehl and Birkle, 2014), or frontal hypoactivation such as Attention-Deficit/Hyperactivity-Disorder (ADHD) (Strehl et al., 2017, Heinrich et al., 2007). Further, significant improvements after SCP neurofeedback were reported for oppositional behavior and physical aggression in children with ADHD (Heinrich et al., 2007), criminal psychopathy (Konicar et al., 2015) as well as for migraine (Siniatchkin et al., 2000). SCP neurofeedback has been considered as the best validated standard neurofeedback protocol approach for ADHD (Arns et al., 2014, Mayer et al., 2013), with stable symptom improvements reported for diverse disorders (Strehl and Birkle, 2014, Gani et al., 2008).

Considering the described reductions in frontal brain activity and cortical excitability linked to an attenuated CNV found in individuals with ASD (Wangler et al., 2011), SCP neurofeedback seems to target a critical neurofunctional aspect of ASD. Regarding brain imaging studies reporting abnormal ACC activation in ASD, together with studies reporting increased ACC activation after SCP training (Gevensleben et al., 2014), SCP neurofeedback appears to be a highly promising non-invasive, non-pharmacological and side-effect-free clinical avenue for the treatment of adolescents with ASD.

Therefore, the main aim of this first clinical SCP trial in ASD is to analyze SCP training effects on the core symptomatology of ASD. A further aim of the current study is to shed light on the full brain training dynamics (regarding task-related changes in SCP but also regarding possible changes in the frequency domain) and overcome traditional (neurofeedback) data analysis pitfalls such as high inter-individual variability, aggregate analyses (Klöbl et al., 2020), multiplicity, and the critically discussed use of *p*-values and arbitrary cut-offs (Cohen, 1994, Wagenmakers et al., 2018). To this aim, we apply Bayesian multi-level statistical techniques and perform hypothesis testing via evidence ratios (ERs).

## 2 Materials and methods

### 2.1 Experimental design

To investigate whether adolescents with ASD can learn to regulate their cortical activity from SCP neurofeedback and whether symptoms of ASD decrease after SCP neurofeedback, a randomized controlled intervention study was conducted (Clinical Trial Registration: DRKS00012339).

### 2.2 Participants

A total of 41 adolescents with a diagnosis of ASD based on state-of-the-art diagnostic instruments (ADI-R (Bölte et al., 2006); ADOS-2 (Poustka et al., 2015)) between the age of 12–17 years (mean age of final sample: 14.05 years; SD = 1.76; all right handed; male) were recruited. Participants with an IQ below 70 (HAWIK-IV (Petermann and Petermann, 2013)), or neurological and medical conditions which render the implementation of EEG/MRI measurements or neurofeedback training impossible (i.e., head injuries, major axis I diagnosis of psychosis, obsessive–compulsive disorder, severe motor or vocal tics, Tourette syndrome, or severe depression with suicidality) were excluded. In addition, participants with simultaneous participation in a pharmacological study or former neurofeedback training experience were also excluded from the study. Accompanying psychopharmacological or psychosocial interventions were permitted throughout the study, but had to be kept constant during the four weeks before and until the end of the study. Written informed consent was obtained from all participants and their parents or legal guardian before being enrolled. The study was approved by the Ethics Committee of the Medical University of Vienna and conducted according to the Declaration of Helsinki.

### 2.3 Experimental procedure

**Fig. 1:**
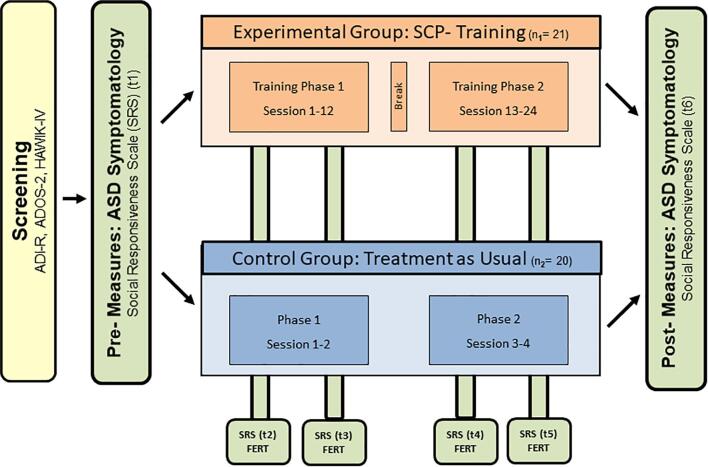
Study overview. Screening (including a diagnosis of ASD, IQ assessment and inclusion/exclusion criteria check), interventions in experimental and control condition, as well as pre-, post- and intervention-accompanying measures. In addition, on an exploratory basis, we assessed general mood, levels of motivation, concentration, fun, goal attainment, arousal and well-being (before every SCP training) as well as possible influences regarding treatment-related trainer and participant variables (4 times in the control- and 6 times in the experimental group via FERT (Klimesch et al., 2007); for details see SI (D), (E)). ADI-R: Diagnostic Interview for Autism-Revised ADOS-2: Diagnostic Observation Schedule for Autistic Disorders 2 FERT: Fragebogen zur Erfassung relevanter Therapiebedingungen (questionnaire for the assessment of therapy conditions) HAWIK IV: Hamburg-Wechsler-Intelligence Test for children and youth.

After screening, participants were randomly allocated to either an experimental group (n_1_ = 21), receiving 24 sessions of EEG-based brain regulation training or to an active control group (n_2_ = 20), which received the conventional treatment (treatment as usual, e.g., clinical counseling), and were informed in detail about the further procedure of the following 3 months intervention period at the Department of Child and Adolescent Psychiatry at the Medical University of Vienna (Fig. 1).

#### 2.3.1 Experimental Group: Neurofeedback training of Slow Cortical Potentials

All participants in the experimental group underwent 24 SCP training sessions, subdivided into two training phases (each comprising 12 training sessions) including a one-week training break between the two training phases. During this 7-days break, participants were asked to exercise their individual training strategies at home without technical support, using a small reminder card showing their preferred training object that was also displayed on the screen during the training. This was done in order to encourage the transfer and generalization of the learned skills in everyday life. In addition, each participant received a structured home training diary in which to record the completed exercises at home and document mental strategies, training situations, as well as behavioral changes during the break.

**Fig. 2:**
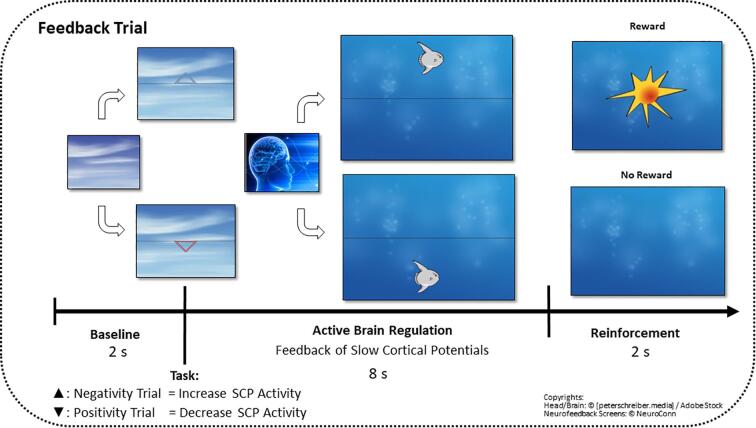
The SCP neurofeedback setting. At the beginning of each trial, a triangle was displayed, specifying the polarity of the requested SCP shift of the upcoming regulation trial: a triangle pointing upwards required negative SCP shifts (increase of cortical activation), while a triangle downwards indicated required positive SCP shifts (inhibition of cortical activation). After baseline recording, the current SCP activity was displayed as an object (e.g., a fish or a moon) at the participants’ screen in real time and moved accordingly to the participants’ brain activity upwards (indicating an increase in cortical activation) or downwards (indicating a decrease in cortical activation). The participants should learn how to volitionally move the object up or down by controlling their SCP in the required polarity. All successful changes (i.e., declinations from baseline in the required polarity with duration of 2 consecutive sec in the last 4sec of each trial) were rewarded with the symbol of a sun after each trial and motivational feedback from trainers.

SCP brain activity was recorded from fronto-central brain areas and presented on the participants’ monitor via a graphical object (see Fig. 2 for a description of the SCP training procedure). Each active regulation phase of one SCP training session consisted of 120 trials, divided into 3 training blocks (8 min each block) with different conditions: the first and the last training block were *feedback conditions* (contingent SCP activity was displayed at the participants’ monitor), whereas the middle training block was a *transfer condition* without the presentation of brain activity on the computer screen. In accordance with established neurofeedback training protocols in ADHD (Strehl et al., 2017), the first aim was a general learning of the volitional modification of brain states (required negativity 50%, required positivity 50%) and after the training break the training aimed for disorder specific regulation (required negativity 80%, required positivity 20%). Within the training blocks, the tasks (required negativity/ positivity) were presented in random order.

#### 2.3.2 Control group: Treatment as usual

Parallel to the intervention phase in the experimental group, participants in the control group received clinical counseling as the treatment as usual (TAU) of the Department of Child and Adolescent Psychiatry at the Medical University of Vienna. Each participant underwent four one-hour appointments during the whole study phase, which were scheduled in equal time intervals (see Fig. 1). During these visits with psychologists and/or medical doctors, the general well being as well as developmental steps and disorder-related deficits were tracked and discussed. Based on the diagnostic procedure and disorder-specific observations, helpful strategies for daily life (e.g., optimize daily duties like homework, cleaning the room, etc.) were offered. At the end of the study, a person- and symptom-specific intervention recommendation was communicated to the parents and further reading materials were provided.

### 2.4 Materials

#### 2.4.1 Psychological measures - Social Responsiveness Scale

To measure autistic traits, we used the Social Responsiveness Scale (SRS (Poustka and Bölte, 2008), a well-validated parent/teacher report to assess deficits in reciprocal social behavior. The questionnaire comprises five subscales: Social Awareness *(SA),* Social Cognition *(SCOG),* Social Communication *(SCOM)*, Social Motivation *(SMOT)* and Restricted Interests and Repetitive Behavior *(Autistic Mannerism; AM)* and a total score representing an index of deficiency in social communication and interaction can be computed. The parents or legal guardians of the experimental, as well as of the control group were asked to fill out the SRS at six time points (pre and post intervention as well as four times during the interventions), in equal time intervals (see Fig. 1).

#### 2.4.2 Neural measures - Slow Cortical Potential training: EEG recording and processing

SCP activity was measured at EEG electrode site FCz. The left mastoid was used as a reference and the right mastoid as ground. Ocular activity was measured by placing electrodes above and below the right eye for blinks and vertical eye movements and at the outer canthi for horizontal eye movements. Electrode impedances were kept below 3 kOhm throughout the study. The signals were recorded with a sampling rate of 128 Hz and with a 40 Hz low-pass online filter. EEG artifacts were detected automatically during the SCP training by the Theraprax System (NeuroConn GmbH, Germany) using a movement artifact correction (Schlegelmilch et al., 2004). Correspondingly, trials containing artifacts were interrupted and repeated.

All offline EEG preprocessing steps were performed using MNE-Python (Gramfort, et al., 2014). A zero-phase finite impulse response (FIR) filter with passband frequency 0.01-2 Hz was used for filtering the raw data. Upon filtering, EEG data for each participant in each *Session* (session 1–24), each *Condition* (feedback 1, transfer, feedback 2), and each *Task* (required positivity, negativity) were segmented into epochs consisting of a 2 seconds baseline window and an 8 seconds trial window. Epochs were then baseline corrected by subtracting the mean of the 2 seconds baseline window from the trial window. Only the last 4 seconds of each regulation trial were used for SCP amplitudes to exclude influences of early event-related potentials on the SCP data (in line with previous worke.g (Strehl et al., 2017). Trials with further artefacts were detected and removed using the *autoreject* algorithm (Jas et al., 2017). The algorithm determines an amplitude rejection threshold adaptively for each participant in each session instead of relying on a global threshold for the entire sample. Epochs which remained after the artifact rejection step were averaged within each session, feedback condition and task.

For the statistical analysis of the signal in the *time domain*, EEG activity in the last 4 seconds of the averaged epochs was averaged once again to obtain a single numerical value representing the mean SCP amplitude.

For the statistical analysis of the signal in the *frequency domain*, power spectral density (PSD) values from the last 4 seconds of each epoch were computed using the multitaper method (Percival DB, Walden AT. Spectral Analysis for Physical Applications. Cambridge University Press;, 1993). The multitaper method was applied to each epoch with a default window half-bandwidth of 4, using adaptive weights and only tapers with >90% spectral concentration within a given bandwidth (low bias). Frequency bands of interest were delta (0.5-4 Hz), theta (4-8 Hz) and alpha (8-12 Hz). PSD values in each frequency band were averaged across epochs separately for each participant, session, feedback condition, and task. The FIR filter bandpass frequency was changed to 0.01-40 Hz, otherwise the same preprocessing steps as in the SCP analysis were employed.

Abnormally large amplitudes or PSD measures were classified as extreme values via Tukey’s boxplot method and removed from subsequent analyses.

### 2.5 Statistical analysis

#### 2.5.1 Bayesian multilevel modeling

The current SCP and psychological data (SRS) represent repeated measurements nested within participants. We therefore applied a multilevel modeling approach because it offers a natural way to analyze such data by accounting for and quantifying inter-individual variability in regression coefficients (Gelman et al., 2013). This encoded our assumption that participants might exhibit different baselines, SCP learning curves or behavioral changes.

To quantify the uncertainty in our estimates in a principled way, we opted for a Bayesian treatment of the multilevel framework. With a Bayesian approach, we can provide a full distribution over plausible parameter values instead of mere point estimates. We then used the *posterior distribution,* containing the entire information about a parameter, to extract credible intervals and test relevant hypotheses regarding the numerical values of the parameters.

We also investigated how well different models encoding varying assumptions about the temporal structure of the data (i.e., linear vs. quadratic learning curves) predict the data, trading off their complexity (i.e., penalizing too many parameters). Therefore, we employed the widely applicable information criterion (WAIC) model comparison to decide between two models: a linear and a quadratic model of SCP and SRS data. For all subsequent analyses, we used the *R*-package *brms f*or fitting and comparing Bayesian multilevel models (Bürkner, 2017). For each of the models considered in this work, we confirmed convergence of the Markov chains for each model parameter via visual inspection and inspection of the Gelman-Rubin convergence metric $\hat{R}$.

#### 2.5.2 Psychological data - Social Responsiveness Scale:

The total score of the Social Responsiveness Scale (SRS (Poustka and Bölte, 2008)) was used as an indicator of the general severity of ASD-related deficits and used as an outcome in univariate multilevel model. At a more fine-grained level, we analyzed changes in the five separate subscales. In order to represent residual correlations and correlations between varying effects across subscales within a single joint model, we further fit a multivariate multilevel model with the individual subscales as a multivariate outcome. A multivariate model is an adequate choice in this context, since it can improve shrinkage effects and account for interdependencies between the different subscales. We defined the following two regression models, both containing the predictors *Group* (Control group vs. Experimental group) and *Time of Measurement* (t_1_, t_2_, t_3_, t_4_, t_5,_ t_6_). We also allow varying intercepts and slopes across participants:

$M_{1}^{\mathrm{SRS}}$: linear change with random intercepts and linear terms across participants, or using Wilkinson notation:

$$\mathrm{Score}=Group*Time+\left(1+Time\right|\mathrm{Person})$$

$M_{2}^{\mathrm{SRS}}$: quadratic change with random intercepts, linear, and quadratic terms across participants, i.e., using Wilkinson notation:

$$\mathrm{Score}=Group*\left(Time+{\mathrm{Time}}^{2}\right)+\left(1+Time+{\mathrm{Time}}^{2}\right|\mathrm{Person})$$

To perform model selection, we computed the *WAIC* metric for the two models and, based on the *WAIC*, we derived model weights to ease comparison (McElreath, 2020). Accordingly, a weight is assigned to each model encoding its relative plausibility. The larger the weight, the better the model performs in terms of the predictive performance/complexity trade-off.

In order to test whether the decline in the SRS scores was stronger in the experimental group than in the control group, we derived a *mean improvement score* (for the total score and all subscale scores) from the model by subtracting the model’s predictions at $t_{6}$ from the prediction at $t_{1}$. Thus, a positive mean improvement indicates a reduction in ASD-related deficits. To quantify evidence that the mean improvement in the intervention group is larger compared to the mean improvement in the control group, we calculated ERs for group differences in total score and the subscale scores of the SRS.

To further ease relevant group comparisons, we computed a difference in mean improvement *(D)* by subtracting the mean improvement of the control group from the mean improvement of the intervention group. We then tested the hypotheses that the *D* values are positive for all SRS scores (indicating superiority of the experimental group).

#### 2.5.3 Neural data - Slow Cortical Potentials Cor P

The linear and the quadratic model are used to describe the learning curves of SCP amplitudes across the factors *Session* (1–24), *Condition* (feedback 1, transfer, feedback 2) and *Task* (negativity, positivity), as well as all interactions between them. We also allowed varying intercepts and slopes across participants and tasks, encoding the assumption that each participant might exhibit a different training process depending on whether the *task* is to produce *negativity* or *positivity*. We defined and fit the following two regression models:

$M_{1}^{\mathrm{SCP}}:$ linear model with random intercepts and linear terms across participants:

Amplitude=Condition∗Task∗Session+(1+Session∗Task|Person)

$M_{2}^{\mathrm{SCP}}$: quadratic model with random intercepts, linear, and quadratic terms across participants:

$$\mathrm{Amplitude}=Condition*Task*\left(Session+{\mathrm{Session}}^{2}\right)+\left(1+\left(Session+{\mathrm{Session}}^{2}\right)*Task|Person\right)$$

As with the SRS data, we performed model selection based on the *WAIC* metric*.*

#### 2.5.4 Neural data – Power Spectral Density

The same hierarchical Bayesian models formulated for the SCP time-domain data were also fitted to the frequency-domain data, with PSD instead of SCP amplitude as an outcome. Separate linear and quadratic models were applied to each of the three frequency bands of interest:

$M_{1}^{\mathrm{PSD}}:$ linear model with random intercepts and linear terms across participants:

PSD=Condition∗Task∗Session+(1+Session∗Task|Person)

$M_{2}^{\mathrm{PSD}}$: quadratic model with random intercepts, linear, and quadratic terms across participants:

$$\mathrm{PSD}=Condition*Task*\left(Session+{\mathrm{Session}}^{2}\right)+\left(1+\left(Session+{\mathrm{Session}}^{2}\right)*Task|Person\right)$$

#### 2.5.5 Hypothesis testing

Following the recommendations of Cohen (Cohen, 1994), we refrain from using arbitrary cut-offs for determining the *significance of an effect* and instead report the actual posterior probability of the hypotheses given the observed data. We interpret the obtained ERs the same way Bayes factors would be interpreted, with qualitative *strength of evidence* labels given according to Kass and Raftery (Kass and Raftery, 1995)and employed only as a communicative heuristic.

## 3 Results

### 3.1 ASD symptomatology – Social Responsiveness Scale

**Fig. 3:**
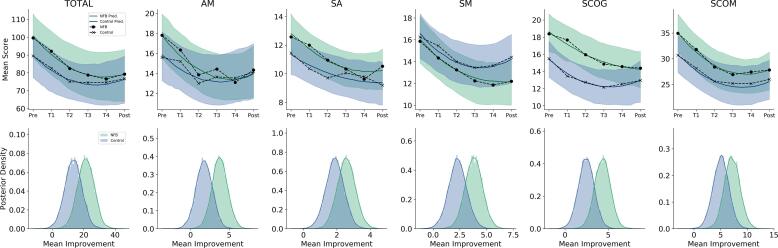
**Social Responsiveness Scale results from the quadratic model.** The first row depicts the model’s mean predictions for each subscale along with 95% credible intervals (shaded regions). Solid black lines depict the empirical means on each occasion. The second row depicts the posteriors of the mean improvements in each group defined as the predicted mean score at $t_{1}$ minus the predicted mean score at $t_{6}$ of a given subscale. NFB = Experimental Neurofeedback Group.

Bayesian model comparison based on the two models’ *WAIC* metrics revealed a preference for the quadratic model, with a relative model weight >0.999 (see **SI (F)** for full model summaries). The high relative model weight indicates that the data of the SRS favors the quadratic model decisively. Indeed, the first row in Fig. 3 depicts the quadratic model’s predictions vs. the empirical means and we observe a strong quadratic decreasing trend both for the SRS total score as well as for each subscale score of the SRS. Moreover, the models’ mean predictions closely match the empirical means (see Fig. 3 and SI (H) for posterior predictive checks). It is also evident that SRS scores declined both in the intervention and in the control group throughout the course of the study.

Further, evidence ratios for the total SRS score as well as for the subscales of the SRS (derived from the quadratic models) are listed in Table 1 indicating support for the superiority of improvement in the neurofeedback treatment group compared to the control group in terms of ASD-related symptom reduction.

**Table 1:** Evidence ratios of SRS scores regarding differences in improvements between groups.

SRS	Difference in Improvement D (95% CI)	Hypothesis	Evidence Ratio	Strength of Evidence
Total	7.88 (-4.58 – 20.19)	*D* > 0	5.79	Substantial
SCOG	1.88 (-0.26 – 4.03)	*D* > 0	12.51	Strong
AM	2.01 (-0.39 – 4.4)	*D* > 0	10.68	Strong
SM	1.51 (-0.48 – 3.45)	*D* > 0	8.86	Substantial
SCOM	2.1 (-1.24 – 5.53)	*D* > 0	5.53	Substantial
SA	0.68 (-0.57 – 1.95)	*D* > 0	4.46	Substantial

Note: Evidence ratios > 1 indicate support for the hypothesis that mean improvement is greater in the experimental SCP-training group than in the control group. Evidence ratios < 1 indicate support for the hypothesis that mean improvement in the control group is greater than or equal to that of the SCP training group. A 95% credible interval (95% CI) gives a numerical range in which the value of the mean difference in improvement falls with a probability of 0.95.

Accordingly, the most difference in improvement is evident for the subscale *Social Cognition (SCOG)* and *Autistic Mannerism (AM)*, followed by improvements in the subscales *Social Motivation (SM)*, *Social Communication (SCOM)*, and *Social Affect (SA)*. A look at the full posterior variability (uncertainty) in the model estimates of mean improvement (plotted in the second row of Fig. 3) suggests that the relatively small evidence ratios are due to the uncertainty owing to the small sample size in both groups.

However, mean differences in improvement are still clearly detectable, corroborating the numerical ranges of the evidence ratios in our population of adolescents with ASD. On a more practical, clinical side, both groups exhibited a relatively large reduction in SRS scores during the course of the study. The total mean improvement was marked by a reduction of 21.38 (*SD* = 5.29) SRS points in the intervention group and 13.5 (*SD* = 5.44) SRS points in the control group.

### 3.2 Slow Cortical Potential neurofeedback training

**Fig. 4:**
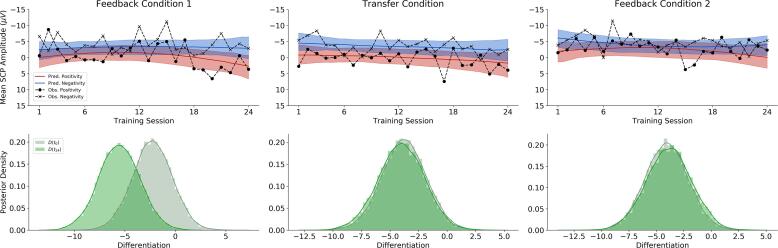
**SCP neurofeedback results from the quadratic model.** The first row depicts the quadratic model’s mean predictions vs. empirical means across all sessions for each *Feedback condition* × *Task* combination. The shaded regions indicate 95% posterior credible intervals. The marked dashed lines indicate the empirical means in each session, condition and task. The second row depicts the posteriors of differentiation (negativity – positivity) at the beginning of training, (*D*($t_{1}$), light green) and at the end of training (*D*(t_24_), dark green). (For interpretation of the references to colour in this figure legend, the reader is referred to the web version of this article.)

Bayesian model comparison revealed that SCP neurofeedback data suggest a slight preference for the quadratic model compared to the linear model in terms of data fit/complexity trade-off, with a relative weight of 0.62 (see SI (F), (H) for detailed model results and posterior predictive checks). The mean predictions of the quadratic model as well as the empirical session means are displayed in the first row of Fig. 4. We observe noticeable multilevel shrinkage due to the small sample size and the large variability in the data. Moreover, the first feedback condition showed the most pronounced differentiation.

**Fig. 5:**
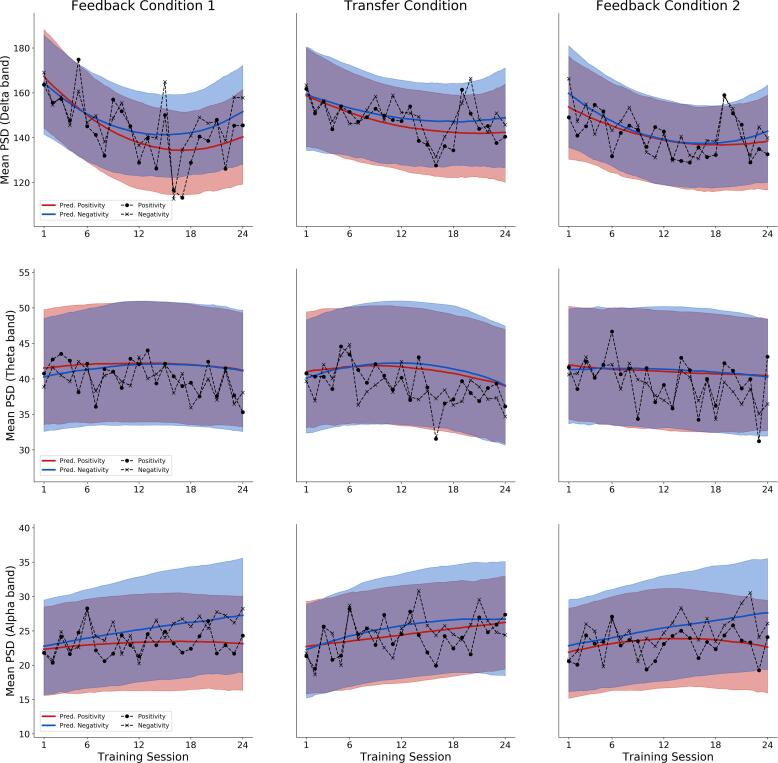
Task-related Power Spectral Density predictions from the quadratic models. The first row depicts the quadratic model’s mean predictions of mean PSD for the delta frequency band, the second row for the theta frequency band and the third row for the alpha frequency band. Predicted and empirical means are depicted across all sessions for each *Feedback condition* × *Task* combination. The shaded regions indicate 95% posterior credibility intervals. The marked dashed lines indicate the empirical means in each session, condition and task.

Regarding the first training block (feedback 1 condition), a pronounced increase in differentiation (absolute difference between brain activity in negativity trials and brain activity in positivity trials) of around 3 µV from the beginning (−2.28 µV, 95% CI: [−6.15 µV–1.54 µV]) until the end (−5.54 µV, 95%CI: [−9.55 µV−1.53 µV]) of the training was achieved by the participants, as depicted in Fig. 4, Fig. 5.

In contrast, during the whole course of block 2 (transfer condition), the differentiation between the two brain states appears all the time pronounced (with just a slight increase of 0.25 µV), starting with -3.77 µV (95% CI: [-7.61 µV – 0.05 µV]) and ending with of -4.01 µV (95% CI: [-8.05 µV – -0.15 µV]) of absolute SCP differentiation in the transfer condition.

Finally, the third training block (feedback 2 condition) reveals an onset pattern similar to that of the transfer condition, but yet a third pattern regarding the further course. Here, the SCP differentiation start from −4.08 µV (95%CI: [−7.95 µV – −0.23 µV]) at the beginning of the training in condition 2, and slightly decreases (0.14 µV) to −3.94 µV (95%CI: [−7.88 µV – 0.13 µV] at the end.

The second row in Fig. 4 illustrates the differences in differentiation between the positivity task and the negativity task via *difference posteriors* (i.e., differences between the posterior distributions at the end and at the beginning of training). While differentiation in the first feedback condition was noticeably stronger at the end of the training compared to the onset of training (first column), no substantial differentiation could be seen in the difference posteriors obtained from the transfer and second feedback condition (second and third columns).

In order to test how plausible these differences (and lack of differences) in differentiation are, we computed ERs for the hypothesis that differentiation is stronger at the end, compared to the beginning of training. The evidence ratios for each condition are listed in Table 2**.** We tested this hypothesis for each condition by considering the difference posteriors depicted in the third row of Fig. 4. The strongest support was found for the first feedback condition with an ER of about 13, whereas for the other conditions only negligible changes in differentiation are present. These ERs support the pattern revealed by the difference posteriors (see second row of Fig. 4). Mean SCP- activity averaged over six successive sessions for each condition and regulation task is presented in Fig. 1**,** SI (B)**.**

**Table 2:** SCP differentiation results for each training condition.

Block	Difference in Improvement D (95% CI)	Hypothesis	Evidence Ratio	Strength of Evidence
Feedback 1	−3.26 (-6.9 – 0.4)	*D* < 0	13.2	Strong
Transfer	−0.24 (-3.88 – 3.4)	*D* < 0	1.19	Barely worth mentioning
Feedback 2	0.24 (-3.46 – 3.94)	*D* < 0	0.84	Negative

Note: Evidence ratios > 1 indicate support for the hypothesis that differentiation is greater at the last occasion compared to first occasion. Evidence ratios < 1 indicate support for the hypothesis that differentiation is smaller at the last occasion compared to the first occasion. A 95% credible interval (95%-CI) gives a numerical range in which the value of the mean difference in differentiation falls with a probability of 0.95.

Explorative analysis regarding relationships between the SRS and SCP data are deposited and discussed in SI (G).

### 3.3 Power spectral density

Bayesian model comparison revealed a clear preference for a quadratic model over a linear model for each of the three frequency bands of interest.^1^ The averaged PSD values and the corresponding predictions of the quadratic models for each frequency band are provided in Fig. 5 (see also SI (F), (H) for detailed model results and posterior predictive checks). These exploratory results reveal a rather complex pattern of change across sessions, feedback conditions and frequency bands.

Regarding delta frequency band power, a decrease from the beginning of the training towards the end of the first training phase (session 1–12) could be observed. The second training phase displays a slight increase in mean PSD of the Delta band again (slight U-shape progression), which ends in session 24 below the initial PSD values, but with an upward trend. Since the same pattern is present in both the negativity and positivity tasks, it is suggestive of on overall decrease in delta frequency band power. However, delta band PSD values in the positivity task decrease faster and remain consistently lower than those in the negativity task. Moreover, this pattern persists across feedback conditions, but appears most pronounced in the first feedback condition.

The model-based analysis of mean PSD in theta frequency band over time exhibits neither remarkable progress or change over time, nor remarkable differences between the different tasks.

Finally, a slight increase in alpha power is observable in all feedback blocks throughout training, opposite to the decrease in delta band power. Regarding the two different tasks, an increasing differentiation in alpha power between the positivity task and the negativity tasks is visible in all three blocks, most pronounced in training blocks 1 und 3 (the feedback conditions).

Since these analyses are exploratory and the authors had no prior expectations regarding changes in PSD, no evidence ratios or correlations with behavioral variables are reported.

## 4 Discussion

The current study investigated the effects of SCP training on core symptomatology in adolescents with ASD. The analysis was based on Bayesian multilevel modeling techniques in order to obtain a more fine-grained analysis of neuroregulatory and behavioral changes throughout the entire course of the training.

Our results revealed that ASD-symptoms improved substantially in both study groups. This improvement was more pronounced in the SCP neurofeedback training group than in the control group. Besides a reduction in the general severity of ASD symptomatology indicated by a decrease in *SRS Total* score (i.e., a general improvement of social responsivity), the subscales *Social Cognition* and *Autistic Mannerism* were those that differentiated the most between the experimental and the control group. Although random differences between the SRS scores of the groups, present at the beginning of the interventions, may limit straightforward interpretations of the treatment improvements, numerous previous SCP studies support our findings of disorder-specific improvements related to SCP neurofeedback training (Strehl et al., 2017, Heinrich et al., 2007, Konicar et al., 2015, Siniatchkin et al., 2000, Mayer et al., 2013, Gani et al., 2008, Wangler et al., 2011).

Additionally, we observed a quadratic reduction in ASD symptomatology in both groups. This result has implications for future treatment approaches, as it can shape expectations about symptom changes during the course of treatment. Moreover, a quadratic symptom decrease implies that the benefits of neurofeedback training are expected to flatten out at some point during training. At this point, further training would elicit no additional improvements in terms of the particular psychopathological outcome. This suggests a consolidation of the learned differentiation with consequent stability and no further improvement upon reaching a plateau.

The additional analyses of potential changes in PSD in the delta frequency band revealed a decrease over time and an enhanced differentiation between the neurofeedback tasks (negativity vs positivity). This preliminary finding indicates a possible balancing effect of SCP training (i.e., a reduction of the often reported slowing of oscillations in the frontal cortex (Kouijzer et al., 2009, Wang et al., 2013) in adolescents with ASD. Moreover, our analysis revealed an increase in alpha frequency power over the whole course of SCP-training. As the current analysis of PSD is preliminary, further research is needed to evaluate if an increase in alpha frequency power could be linked to improved inhibition (Klimesch et al., 2007, Weisz et al., 2011) and improved executive functions, both of which have been found to be disrupted in individuals with ASD (Yuk et al., 2020) In the same vain, the effects of alpha band activity, arising from long-range communication between different brain regions (Von Stein and Sarnthein, 2000) and a corresponding alpha power increase after neurofeedback training should further be investigated to shed light on the inter-regional connectivity and diminished long-range coherence found in individuals with ASD (Wang et al., 2013).

Besides the clinical improvement of ASD psychopathology, another aim of the current study was to pave the road for an optimized neurofeedback methodology and protocol applicable to individuals with ASD. To this aim, we analyzed the entire process of SCP brain regulation via Bayesian multilevel methods. Our analyses revealed that a quadratic multilevel model provides a better description of the SCP training process than a linear model. The presented findings reveal the complexity and non-linearity of the SCP neurofeedback training process and have implications for future analyses of neurofeedback data as well as regarding the critical discussion of training protocols. Furthermore, our results highlight the importance of analyzing the entire training process (for in-depth discussions of the different conditions and tasks, as well as related future recommendations see SI (B)).

Although the complexity and large variability inherent in the data preclude a decisive interpretation, the results, especially in feedback 1 (first training block) suggest that adolescents with ASD can successfully learn to modulate their brain activity, as measured by the SCP differentiation index.

The observed effects of improved volitional control of prefrontal activity through SCP-training based on an operant conditioning paradigm could possibly indicate neuroplastic changes (Birbaumer et al., 1990) and be linked to a two-strategy mechanism, adjustable to the requirements of the specific situation: Firstly, especially the increased ability to produce negative SCP shifts might be used for increasing and allocating attention and improving executive functions, linked to improved attentional gating in fronto-thalamic-cingulate networks (perhaps reflected also in the improvements of the SRS subscale *Social Cognition)*. Secondly, the remarkable facilitation of the generation of positive SCP shifts in the current study might be used to control excessive arousal or regulate inflexible, stereotypic behavior, fostering an improved behavioral regulation (possibly linked to the improved SRS subscale *Autistic Mannerism* with its regulation of inflexible, stereotypic behavior). Considering similarities between ADHD and ASD, such as the often-reported frontal hypoarousal or the dysfunctional ACC (Chan et al., 2011, Craig et al., 2016), the self-inflated change of neural excitation through SCP-regulation, seem to have many benefits compared to conventional treatment by enabling the participants to utilize their regulation skills in every necessary situation adaptively and without high costs or side effects.

Notwithstanding mostly complying with the CRED-nf checklist (Ros et al., 2020) (see SI (A) for checklist) and considering the promising results, the current study has several limitations and cannot provide complete unbiased evidence regarding efficacy and specificity. First, the experimental design of clinical neurofeedback studies poses multiple challenges (e.g., regarding optimal control group/ -sham feedback conditions (Van Dongen-Boomsma et al., 2013)). While practical and ethical advantages of active control groups in adolescents are obvious, still unspecific effects such as the different frequency of interaction with clinical staff, the different settings, possible different expectations and randomly different baseline SRS scores between the groups could not completely be ruled out with such a design. Therefore, future research with more complex experimental designs, including higher samples sizes (feasible in multicenter studies) is needed.

Our exploratory assessment of possible influences of diverse treatment-related trainer and participant variables (via FERT (Vollmann, 2009)), see SI (E)+(D) suggested a constant stability of those nonspecific-effects variables during the course of interventions as no remarkable increase or decrease could be observed for any treatment-related variable; neither in the experimental- nor in the control condition.

In conclusion, this study is the first to demonstrate pronounced ASD-specific improvements, especially in social cognition and motivation, following intensive neurofeedback training of SCPs. Therefore, the volitional modification of SCP presents itself as a neurophysiologically plausible, noninvasive technique for application in clinical settings, and/or adjunct to psychopharmacological and behavioral interventions, if effective, refining treatment approaches for ASD.

## Ethics statement

This study was approved by the Ethics Committee of the Medical University of Vienna, Austria and conducted according to the Declaration of Helsinki. Written informed consent was obtained of all participants and their caregivers before being involved in the study.

## Funding & competing financial interests

This project was funded by the Austrian Science Fund (FWF): KLI600B27.

## Author contributions

L.K., N.B., R.L. and L.P. conceived and designed the study, L.K., K.P., R.D. and M.K. conducted the measurements, L.K. and S.R. analyzed the data, L.K., S.R., P.P., R.L. L.P. and N.B. interpreted the data, L.K., S.R., K.P., M.K., R.D., N.B., R.L., P.P. and L.P wrote, edited and revised the paper. All authors contributed to the preparation of the paper and approved the final manuscript.

## Competing interests

The other authors declare no competing interests.

## Footnotes

1 Leave-One-Out Cross-Validation (LOO-CV) was used to perform Bayesian model comparison, as the approximation of WAIC was unreliable for the models of PSD change (as indicated by the *p_waic* self-diagnostic).

## References

[b0005] Ahmed S.P., Bittencourt-Hewitt A., Sebastian C.L. (2015). Neurocognitive bases of emotion regulation development in adolescence. Dev. Cogn. Neurosci..

[b0010] Arns, M., Heinrich, H., Strehl, U. 2014. Evaluation of neurofeedback in ADHD: The long and winding road. Published online. 10.1016/j.biopsycho.2013.11.013. 24321363

[b0015] Birbaumer N., Elbert T., Canavan A.G.M., Rockstroh B. (1990). Slow potentials of the cerebral cortex and behavior. Physiol. Rev..

[b0020] Bölte S., Rühl D., Schmötzer G., Poustka F. (2006). Diagnostisches Interview Für Autismus-Revidiert (ADI-R).

[b0025] Bürkner P.C. (2017). brms: An R package for Bayesian multilevel models using Stan. J. Stat. Softw..

[b0030] Carrick F.R., Pagnacco G., Hankir A. (2018). The treatment of autism spectrum disorder with auditory neurofeedback: A randomized placebo controlled trial using the mente autism device. Front. Neurol..

[b0035] Chan A.S., Han Y.M.Y., Leung W.W.M., Leung C., Wong V.C.N., Cheung M.C. (2011). Abnormalities in the anterior cingulate cortex associated with attentional and inhibitory control deficits: A neurophysiological study on children with autism spectrum disorders. Res. Autism. Spectr. Disord..

[b0040] Coben R., Clarke A.R., Hudspeth W., Barry R.J. (2008). EEG power and coherence in autistic spectrum disorder. Clin. Neurophysiol..

[b0045] Cohen J. (1994). The earth is round (p <.05). Am Psychol..

[b0050] Cowan J, for LM-AM of the A, 1994 undefined. EEG biofeedback for the attention problems of autism: A case study. peakachievement.com. Accessed August 6, 2020. http://www.peakachievement.com/Autism Long Abstract for AAPB, 1994.doc.

[b0055] Craig F., Margari F., Legrottaglie A.R., Palumbi R., de Giambattista C., Margari L. (2016). A review of executive function deficits in autism spectrum disorder and attention-deficit/hyperactivity disorder. Neuropsychiatr. Dis. Treat..

[b0060] Datko M., Pineda J.A., Müller R.A. (2018). Positive effects of neurofeedback on autism symptoms correlate with brain activation during imitation and observation. Eur. J. Neurosci..

[b0065] Ecker C., Bookheimer S.Y., Murphy D.G.M. (2015). Neuroimaging in autism spectrum disorder: Brain structure and function across the lifespan. Lancet Neurol..

[b0070] Ernst M., Pine D.S., Hardin M. (2006). Triadic model of the neurobiology of motivated behavior in adolescence. Psychol. Med..

[b0075] Gani C, Birbaumer N, Strehl U. Long Term Effects after Feedback of Slow Cortical Potentials and of Theta-Beta-Amplitudes in Children with Attention-Deficit/Hyperactivity Disorder (ADHD). Vol 10.; 2008. Accessed August 7, 2020. www.ijbem.org.

[b0080] Gelman A., Carlin J.B., Stern H.S., Dunson D.B., Vehtari A., Rubin D.B. (2013). Bayesian Data Analysis.

[b0085] Gevensleben H., Albrecht B., LÃtcke H. (2014). Neurofeedback of slow cortical potentials: neural mechanisms and feasibility of a placebo-controlled design in healthy adults. Front. Hum. Neurosci..

[b0090] Gramfort A, M L, E L, et al. MNE software for processing MEG and EEG data. Neuroimage. 2014;86:446-460. doi:doi: 10.1016/j.neuroimage.2013.10.027. 10.1016/j.neuroimage.2013.10.027 PMC3930851 24161808

[b0095] Hazlett H.C., Gu H., Munsell B.C. (2017). Early brain development in infants at high risk for autism spectrum disorder. Nature.

[b0100] He B.J., Raichle M.E. (2009). The fMRI signal, slow cortical potential and consciousness. Trends Cogn. Sci..

[b0105] Heinrich H., Gevensleben H., Strehl U. (2007). Annotation: Neurofeedback - Train your brain to train behaviour. J. Child. Psychol. Psychiatry Allied Discip..

[b0110] Holtmann M., Steiner S., Hohmann S., Poustka L., Banaschewski T., Bölte S. (2011). Neurofeedback in autism spectrum disorders. Dev. Med. Child. Neurol..

[b0115] Hoofs V., Princen M.M., Poljac E., Stolk A., Poljac E. (2018). Task switching in autism: An EEG study on intentions and actions. Neuropsychologia.

[b0120] Jas M., Engemann D.A., Bekhti Y., Raimondo F., Gramfort A. (2017). Autoreject: Automated artifact rejection for MEG and EEG data. Neuroimage..

[b0125] Kass R.E., Raftery A.E. (1995). Bayes factors. J. Am. Stat. Assoc..

[b0130] Klimesch W., Sauseng P., Hanslmayr S. (2007). EEG alpha oscillations: The inhibition-timing hypothesis. Brain Res. Rev..

[b0135] Klöbl M., Michenthaler P., Godbersen G.M., Robinson S., Hahn A., Lanzenberger R. (2020). Reinforcement and punishment shape the learning dynamics in fMRI neurofeedback. Front Hum. Neurosci..

[b0140] Konicar L., Veit R., Eisenbarth H. (2015). Brain self-regulation in criminal psychopaths. Sci. Rep..

[b0145] Kouijzer M.E.J., de Moor J.M.H., Gerrits B.J.L., Buitelaar J.K., van Schie H.T. (2009). Long-term effects of neurofeedback treatment in autism. Res. Autism. Spectr. Disord..

[b0150] Kouijzer M.E.J., de Moor J.M.H., Gerrits B.J.L., Congedo M., van Schie H.T. (2009). Neurofeedback improves executive functioning in children with autism spectrum disorders. Res. Autism. Spectr. Disord..

[b0155] Landa R.J. (2018). Efficacy of early interventions for infants and young children with, and at risk for, autism spectrum disorders. Int. Rev.Psychiatry..

[b0160] Mayer K., Wyckoff S.N., Strehl U. (2013). One size fits all? Slow cortical potentials neurofeedback: A review. J. Atten. Disord..

[b0165] McElreath, R., 2018. Statistical Rethinking: A Bayesian Course with Examples in R and Stan. Chapman and Hall/CRC.

[b0175] Percival, D.B., Walden, A.T. 1993. Spectral Analysis for Physical Applications. Cambridge University Press. 10.1017/cbo9780511622762.

[b0180] Petermann, F., Petermann, U. 2013. Wechsler Adult Intelligence Scale. Deutschsprachige Adaptation Der WAIS-IV von D. Wechsler. Pearson Assessement.

[b0185] Pineda J.A., Carrasco K., Datko M., Pillen S., Schalles M. (2014). Neurofeedback training produces normalization in behavioural and electrophysiological measures of high-functioning autism. Philos. Trans. R. Soc. B Biol. Sci..

[b0190] Poustka F., Bölte S. (2008). Skala Zur Erfassung Sozial Reaktivität (SRS).

[b0195] Poustka L., Kamp-Becker I. (2016). Current practice and future avenues in autism therapy. Curr. Topics Behav. Neurosci..

[b0200] Poustka L., Rühl D., Feineis-Matthews S., Poustka F., Hartung M., Bölte S. (2015). ADOS-2. Diagnostische Beobachtungsskala Für Autistische Störungen - 2. Deutschsprachige Fassung Der Autism Diagnostic Observation Schedule.

[b0205] Ros T., Enriquez-Geppert S., Zotev V. (2020). Consensus on the reporting and experimental design of clinical and cognitive-behavioural neurofeedback studies (CRED-nf checklist). Brain.

[b0210] Schlegelmilch F., Markert S., Berkes S., Schellhorn K. (2004). Online ocular artefact removal for DC-EEG-signals: Estimation of DC- Level. Biomed. Tech..

[b0215] Sichel A.G., Fehmi L.G., Goldstein D.M. (1995). Positive outcome with neurofeedback treatment in a case of mild autism. J. Neurother..

[b0220] Siniatchkin M., Hierundar A., Kropp P., Kuhnert R., Gerber W.D., Stephani U. (2000). Self-regulation of slow cortical potentials in children with migraine: An exploratory study. Appl. Psychophysiol. Biofeedback.

[b0225] Strehl U., Birkle S.M., WÃrz S., Kotchoubey B. (2014). Sustained reduction of seizures in patients with intractable epilepsy after self-regulation training of slow cortical potentials â€“ 10 years after. Front. Hum. Neurosci..

[b0230] Strehl U., Aggensteiner P., Wachtlin D. (2017). Neurofeedback of slow cortical potentials in children with attention-deficit/hyperactivity disorder: A multicenter randomized trial controlling for unspecific effects. Front. Hum. Neursci..

[b0235] Van Dongen-Boomsma M., Vollebregt M.A., Slaats-Willemse D., Buitelaar J.K. (2013). A randomized placebo-controlled trial of electroencephalographic (EEG) neurofeedback in children with attention-deficit/hyperactivity disorder. J. Clin. Psychiatry.

[b0240] Vollmann, K. 2009. Entwicklung und Überprüfung eines Fragebogens zur Erfassung relevanter Therapiebedingungen (FERT) – PDF Kostenfreier Download. Published online 2009. Accessed August 10, 2020. https://docplayer.org/122676975-Entwicklung-und-ueberpruefung-eines-fragebogens-zur-erfassung-relevanter-therapiebedingungen-fert.html.

[b0245] Von Stein A., Sarnthein J. (2000). Different frequencies for different scales of cortical integration: From local gamma to long range alpha/theta synchronization. Int. J. Psychophysiol..

[b0250] Wagenmakers E.J., Marsman M., Jamil T. (2018). Bayesian inference for psychology. Part I: Theoretical advantages and practical ramifications. Psychon. Bull Rev..

[b0255] Wang J., Barstein J., Ethridge L.E., Mosconi M.W., Takarae Y., Sweeney J.A. (2013). Resting state EEG abnormalities in autism spectrum disorders. J. Neurodev. Disord..

[b0260] Wangler S., Gevensleben H., Albrecht B. (2011). Neurofeedback in children with ADHD: Specific event-related potential findings of a randomized controlled trial. Clin. Neurophysiol..

[b0265] Weisz N., Hartmann T., Müller N., Lorenz I., Obleser J. (2011). Alpha rhythms in audition: Cognitive and clinical perspectives. Front. Psychol..

[b0170] World Health Organization, 2004‎. ICD-10: international statistical classification of diseases and related health problems: tenth revision, 2nd ed. World Health Organization. https://apps.who.int/iris/handle/10665/42980. 3376487

[b0270] Yuk V., Dunkley B.T., Anagnostou E., Taylor M.J. (2020). Alpha connectivity and inhibitory control in adults with autism spectrum disorder. Mol. Autism..

