# Supplementary material for: Volitional modification of brain activity in adolescents with Autism Spectrum Disorder: A Bayesian analysis of Slow Cortical Potential neurofeedback
Source: Neuroimage Clin. 2021 Jan 9;29:102557. doi: 10.1016/j.nicl.2021.102557 (PMC7829342; doi:10.1016/j.nicl.2021.102557)

**Supplementary Information**

Volitional Modification of Brain Activity

in Adolescents with Autism Spectrum Disorder

A Bayesian Analysis of Slow Cortical Potential Neurofeedback

Konicar, L., Radev, S., Prillinger, K., Klöbl, M., Diehm, R., Birbaumer, N., Lanzenberger, R., Plener, P.L. & Poustka, L.

Contents

| (A) | CRED-nf Checklist | 1 |
| --- | --- | --- |
| (B) | In-depth discussion of the SCP training process | 3 |
| (C) | Regulation Strategies | 9 |
| (D) | General Assessment of Mood, Motivation and Concentration | 10 |
| (E) | Fragebogen zur Erfassung relevanter Therapiebedingungen (FERT) | 13 |
| (F) | Detailed Multilevel Model Results | 15 |
| (G) | Relationships Between SRS and SCP data | 22 |
| (H) | Posterior Predictive Checks | 25 |

(A) CRED-nf Checklist (Ros et al., 2020)

| **Domain** | **Item** | **Checklist item** | **Reported on page #** |
| --- | --- | --- | --- |
| **Pre-experiment** | | |  |
|  | 1a | Pre-register experimental protocol and planned analyses | - |
|  | 1b | Justify sample size | - |
| **Control groups** | | |  |
|  | 2a | Employ control group(s) or control condition(s) | 6-9 |
|  | 2b | When leveraging experimental designs where a double-blind is possible, use a double-blind | - |
|  | 2c | Blind those who rate the outcomes, and when possible, the statisticians involved | - |
|  | 2d | Examine to what extent participants and experimenters remain blinded | - |
|  | 2e | In clinical efficacy studies, employ a standard-of-care intervention group as a benchmark for improvement | 8-9 |
| **Control measures** | | |  |
|  | 3a | Collect data on psychosocial factors | 10, SI(D)(E) |
|  | 3b | Report whether participants were provided with a strategy | SI(C) |
|  | 3c | Report the strategies participants used | SI(C) |
|  | 3d | Report methods used for online-data processing and artifact correction | 10-11 |
|  | 3e | Report condition and group effects for artifacts | - |
| **Feedback specifications** | | |  |
|  | 4a | Report how the online-feature extraction was defined | 10-11 |
|  | 4b | Report and justify the reinforcement schedule | 7 |
|  | 4c | Report the feedback modality and content | 8,9 |
|  | 4d | Collect and report all brain activity variable(s) and/or contrasts used for feedback, as displayed to experimental participants | 8,9,18 |
|  | 4e | Report the hardware and software used | 10,11 |
| **Outcome measures** | | |  |
| Brain | 5a | Report neurofeedback regulation success based on the feedback signal | 18-20 |
|  | 5b | Plot within-session and between-session regulation blocks of feedback variable(s), as well as pre-to-post resting baselines or contrasts | 18-20 |
|  | 5c | Statistically compare the experimental condition/group to the control condition(s)/group(s) (not only each group to baseline measures) | - |
| Behaviour | 6a | Include measures of clinical or behavioural significance, defined a priori, and describe whether they were reached | 15-17, SI(D),(E) |
|  | 6b | Run correlational analyses between regulation success and behavioural outcomes | SI (G) |
| **Data storage** | | |  |
|  | 7a | Upload all materials, analysis scripts, code, and raw data used for analyses, as well as final values, to an open access data repository, when feasible | SI _(end)_ |

**(B) In-depth discussion of the SCP training process in the different feedback conditions and tasks**

At the level of the different conditions, in the current study, the most pronounced regulation success, i.e., SCP differentiation (difference between brain activity in negativity and positivity tasks) was achieved in the first feedback condition (first training block). Inspecting the course of each task (negativity/positivity) separately in the first feedback condition, we observed that the mean amplitude in positivity trials was around zero (0.02 µV, 95% CI: [-2.43 µV – 3.16 µV]) at the beginning of the training, whereas the mean amplitude in negativity trials was already -2.47 µV (95% CI: [-5.02 µV – 0.68 µV]) at the onset of SCP training. In the further course of training, the participants could increase the amplitude (i.e., towards a higher negative amplitude) in negativity tasks slightly to -2.87 µV (95% CI: [-6.49 µV –- 0.41 µV]) at the end of the training. Brain activity in the positivity task proceeded in the first training phase parallel to the brain activity in the negativity task: toward the (task-incorrect) – negative µV range, but increased task-correctly again and pronounced in the second training phase up to 2.73 µV (95% CI: [-0.70 µV – 6.66 µV]) at the end of the training. Moreover, the change in activity in positivity tasks appears to follow a negative quadratic trend, since the amplitudes start increase into the positive µV range after session 12. The participants already seemed to be able to differentiate between the two brain states from the very beginning (mean negativity around -2,5 µV, mean positivity around zero), which could also be the reason for the only small further increase in negativity tasks (compared to the pronounced increase in positivity tasks) in the following training course, or it could be due to excitation effects accompanying the start of a new treatment including a new setting, people and tasks. However, regarding the SCP differentiation, the participants could successfully increase the SCP differentiation even further in feedback 1, most pronounced at the end of the training.

In the transfer condition (second training block), brain activity in the negativity tasks and in the positivity tasks follows the same pattern throughout the whole 24 sessions of SCP training: the brain activity in both tasks decreases slightly and constantly parallel; with the SCP activity in the negativity task ending in -2.39 µV (95% CI: [-5.69 µV – 1.12 µV]) and SCP activity in the positivity task ending in 1.7 µV (95% CI: [-1.69 µV – 6.18 µV]). Regarding this slight drift in both tasks, it seems that the transfer condition was either used to solely focus and increase brain activity in positivity regardless of the negativity task, in which the decrease in SCP activity is just a side-effect of the targeted positivity increase or as a relaxation period (unconsciously because of tiredness and cognitive exhaustion). The latter would be in line with the training experience of participants reporting the transfer condition as the most challenging and exhausting condition and therefore tiring, probably because of the missing feedback object. Even if the SCP activity in both tasks seems to decrease slightly over time, the remarkable large SCP differentiation between the two brain states in this condition, which is already evident at the beginning of the training, remains stable throughout the whole 24 sessions. Because of this, a further broadening of the SCP differentiation in this block is rather impossible in regards of the ceiling effect.

In sum, the lack of an obvious transfer effect could be due to different reasons. Firstly, in regards of the SCP differentiation as the chosen index of learning success, the ceiling effect stemming from the already pronounced differentiation abilities already at the beginning of the training has to be taken into account in view of the missing further improvement in that index over time. Nevertheless, as an ideal SCP differentiation could be described as a maximum difference between brain activity during the negativity task present in the negative µV range and the brain activity during the positivity task present in the positive µV range, the found/described SCP differentiation at the end of the transfer condition with brain activity in the negativity task of -2.39 µV and brain activity in the positivity task of 1.7 µV could be seen as highly desirable. Secondly, another interpretation could target the clinical population of ASD with its inflexibility and importance of rituals and constancy (including the importance of constant feedback for stable regulation abilities), requiring an extended effort (compared to other neurofeedback learning populations) to learn and show the same regulation skills in the transfer condition (missing the constant feedback object) than in the feedback condition. If so, the importance of the transfer condition, as an index of the overall training success (i.e., the transferability of the gained brain regulation abilities from the lab into daily life) would be limited. Hence, other parameters for ‘learning’ or ‘success’ (than the hypothesized, specific SCP differentiation in transfer like in the current analysis) must be investigated to discuss and possibly identify more suitable and valid indices for this population. For sure, a follow up measure of SCP regulation abilities would detect if the regulation abilities remain stable (in vitro). Combined with subjective measures (e.g., psychological and behavioral assessments, in vivo) this additional investigation would provide an excellent insight into the long lasting brain regulation learning (transfer) effects, a great concern of us for future studies.

The learning curves observed in the second feedback condition (third training block) permit multiple interpretations. Inspecting the tasks separately, we observe that at onset, the mean predicted amplitude for negativity tasks as well as for positivity tasks are both in the negative µV range: -6.08 µV (95% CI: [-8.66 µV – 3.23 µV]) in the negativity task and -1.92 µV (95% CI: [-4.68 µV – 1.38 µV]) in the positivity task. Afterwards, the brain activities proceed in opposite directions (i.e., negativity amplitudes became less negative, while positivity amplitudes became more negative) until the end of the first training phase (session 1-12). From session 13 on, SCP amplitudes reverted to their baseline values (meaning the SCP amplitudes in the negativity task showed more negative amplitudes, while amplitudes in the positivity task increased in the positive µV range). Finally, the second feedback condition ends with a mean predicted SCP magnitude of -3.97 µV (95% CI: [-6.85 µV – -0.66 µV]) in the negativity task and mean predicted SCP magnitude of -0.03 µV (95% CI: [-4.37 µV – 3.33 µV]) in the positivity task.

On the one hand, the ability to produce negative SCP shifts seems to diminish from the beginning to the middle of the training, and then it slightly increases again in the second training phase from session 12 to 24. On the other hand, the production of positive SCP shifts in the second feedback condition appears to worsen from the beginning until the middle of the training, but improves again until the end of the training. The first decrease in the production of negative SCP shifts could indicate a habituation to the training setting and/or an increase of tiredness through the first training phase. The increase of negative SCP shifts in the second training phase could mirror a regained motivation because of the end of the training and the last chances to really learn brain self-regulation in this setting is coming closer or regained vigilance and motivation due to the relaxations during the break. The production of positive SCP in feedback 2 in the first training phase could be due to the participants’ motivation to be rewarded as much as possible, with the related psychological and physical excitation accompanying the achievement of success, but making a deactivation or decrease of activations during the positivity tasks nearly impossible. The following improvement in the production of positive SCP in the second feedback condition in the second training phase could also mirror a decrease in subjective expectancies and the accompanying relaxation, which again facilitates the production of positive SCP shifts. Regarding the SCP differentiation, a notably low differentiation in the first training phase could be observed in the beginning. Differently, in the following course from session 13 to 24 displays a regaining or recovering and obvious utilization of gained brain regulation skills could be observed. This increase in SCP differentiation starting again from session 13 on, additionally ends in a desirable differentiation with EEG activity in negativity tasks in the negative µV range and brain activity in positivity tasks in the positive µV range, possibly revealing the effects of motivational factors, as shown in **Fig 1**.

Finally, the mean EEG activity averaged over six successive sessions for each condition and regulation task are presented for visual inspection in **Fig. 1**. Corroborating our quantitative results, the brainwave plots reveal a notable increase in differentiation over the course of training only in the first feedback condition (see first row of **Fig. 1**). Exploratory analysis regarding relationships between SRS and SCP data are reported in **SI (G).**


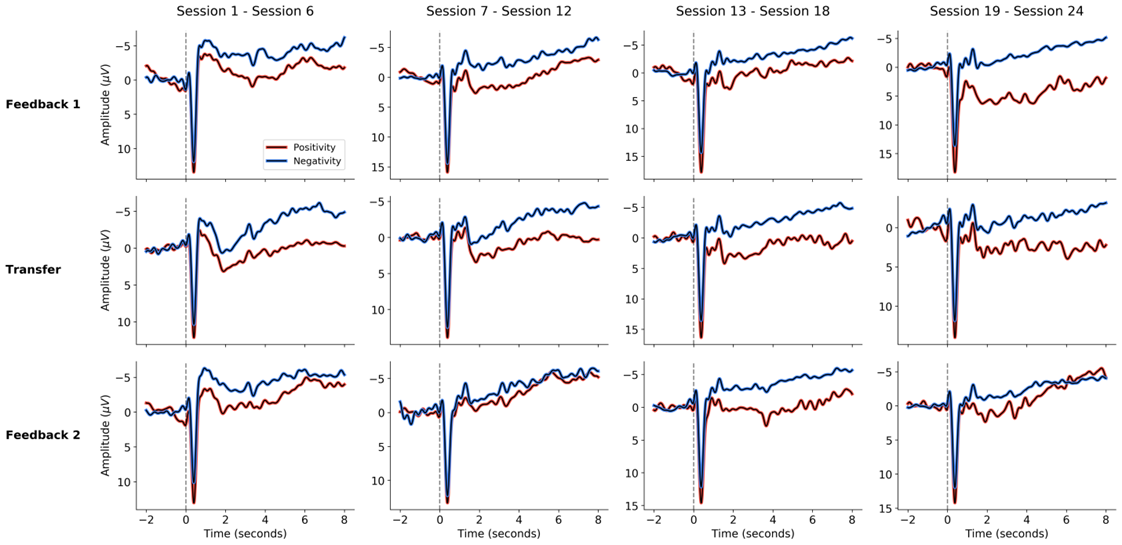


**Figure 1B.** Mean EEG activity averaged over six successive sessions for each condition and regulation task.

The results of the fine-grained analysis of different training conditions and tasks have implications for future neurofeedback training protocols in ASD. Considering the reported findings, a retention or application of the current structure of the SCP training is supported in general.

In view of the structure of the training in total including training phase 1, a break and training phase 2, this conclusion follows observations in feedback 2 that firstly the primary worsening of SCP differentiation in the training phase 1 (session 1-12) could be reversed by the following recurring increasing of SCP differentiation in training phase 2 (session 13-24). Secondly, the SCP differentiations (especially of feedback 1 and the transfer condition) are greater at the end of training phase 2 than at the end of training phase 1. Therefore, a shorter training, e.g., only 12 sessions could lead to a suboptimal training success.

Regarding the structure of one session, the current analysis revealed substantial differences in successful SCP differentiation (in terms of evidence ratios) indicating superior performance in feedback block 1. Although this observation might lead to a recommendation for a reduction of training blocks in future protocols, the characteristics of the specific learning indicator with diverse advantages and disadvantages, as well as the full information of the SCP process provided in Figure 4 have to be taken into account. Despite the fact that the SCP differentiation index remains stable over time in the transfer condition, it changes again in the second feedback block. Here, we observe that differentiation is less pronounced than in the transfer condition, but at the beginning of the intervention, SCP activity in both tasks (negativity and positivity) of feedback block 2 is clearly stronger in the negative µV area than in the transfer condition. This observation could indicate that possibly after the good SCP differentiation increase over time in feedback 1, a relaxing/consolidation phase is needed (transfer condition) for enabling a further increased cognitive performance, required by feedback block 2.

From another perspective, further SCP neurofeedback trials could foster moderating effects of motivational and attentional variables during the transfer block and feedback block 2 by offering longer resting breaks between the different training blocks. A different strategy could be to implement a sophisticated motivational reward system, increasing the quantity and/or quality of reward related to the ongoing duration of the training session. Such a reward strategy should be directly incorporated into the neurofeedback system.

Concerning the sequence of the training blocks, we could only recommend not to position the transfer block at the end of the training due to its challenging and exhausting character, possibly linked to a smaller SCP differentiation in this condition. The finding of the smallest SCP differentiation in the last training block (in the current study a feedback condition) further supports a middle position of the transfer condition.

Regarding the different training tasks, it would be a highly interesting future endeavor to compare a training protocol targeting a two stage approach (like in the current study with same amounts of positivity and negativity tasks in the first training phase and disorder specific distribution in the second training phase) to a training protocol targeting a one stage approach (same amount of positivity and negativity tasks throughout the whole training).

For the possible implementation of the here described recommendation, the multiplicity of possible combinations of the different training protocol parameters (number and sequence of blocks, condition and tasks; selection of feedback signal and measurement position; one or two stage protocol; kind, criteria and amount of reward; recordings of baselines; length of active regulation phase; feedback screens; devices and training settings; different ages, gender and populations of the investigated subjects, etc.) has to be taken into account.

(C) Regulation Strategies

Participants were instructed to move the feedback object according to the task requirements (required production of electrically positive or negative SCP shifts), developing their individual strategy. The instruction emphasized that muscular (i.e., tension-relaxation) or respiratory strategies disturb self-regulation performance. No other instruction was provided regarding the efficiency or the success of specific strategies. The participants’ self-developed strategies were discussed verbally, whenever the participant or the SCP trainers deemed it worthy and necessary, but not assessed following a predefined structure or strategic assessment.

During the training break of one week, participants documented their home training in a structured training diary (3 times a day) in which the used mental regulation strategies (as well as the training situation and times) were documented. Based on those home diary descriptions, we summarize that our sample reported multiple regulation strategies, fitting in the described domains of Hasslinger^1^.

For example, our participants reported strategies ranging from playing computer games to diverse spare time activities including bicycling, skiing or, rollercoaster to strategies such as imagining the fish swimming up/down, calculating/thinking of Pi or imaging the sun. **Table 1B** summarizes the participants’ reported themes in the related domains^1^.

**Table 1B:** SCP Neurofeedback regulation strategies.

| **Domain** | **Themes** | **Total number of entries** |
| --- | --- | --- |
| **Cognitive Domain** | Generating internal phenomena | 78 |
|  | Memory | 13 |
|  | Directed focus | 4 |
|  | Scattered focus | 2 |
|  | Motivation | 1 |
| **Emotional Domain** | Engaging thoughts | 69 |
|  | Specified emotion | 5 |
|  | Mindfulness/relaxation | 3 |
| **Unspecified Domain** | Nothing mentioned | 30 |
|  | Experimenting | 4 |
|  | Passivity | 4 |

(D) General Assessment of Mood, Motivation and Concentration

The exploratory assessment of the general mood and wellbeing indicates that the adolescents of the experimental group indeed enjoyed the intervention (with a decrease at the end of the first training phase and a recurring pleasure with the beginning of the second training phase), whereas the self-assessed goal attainment and the satisfaction with their regulation performance remained stable over the training course. In addition, a trend of a decrease of general arousal, parallel to a trend for an increase in self-reported concentration and wellbeing was displayed by the exploratory assessment of the general mood and well-being in the experimental condition.


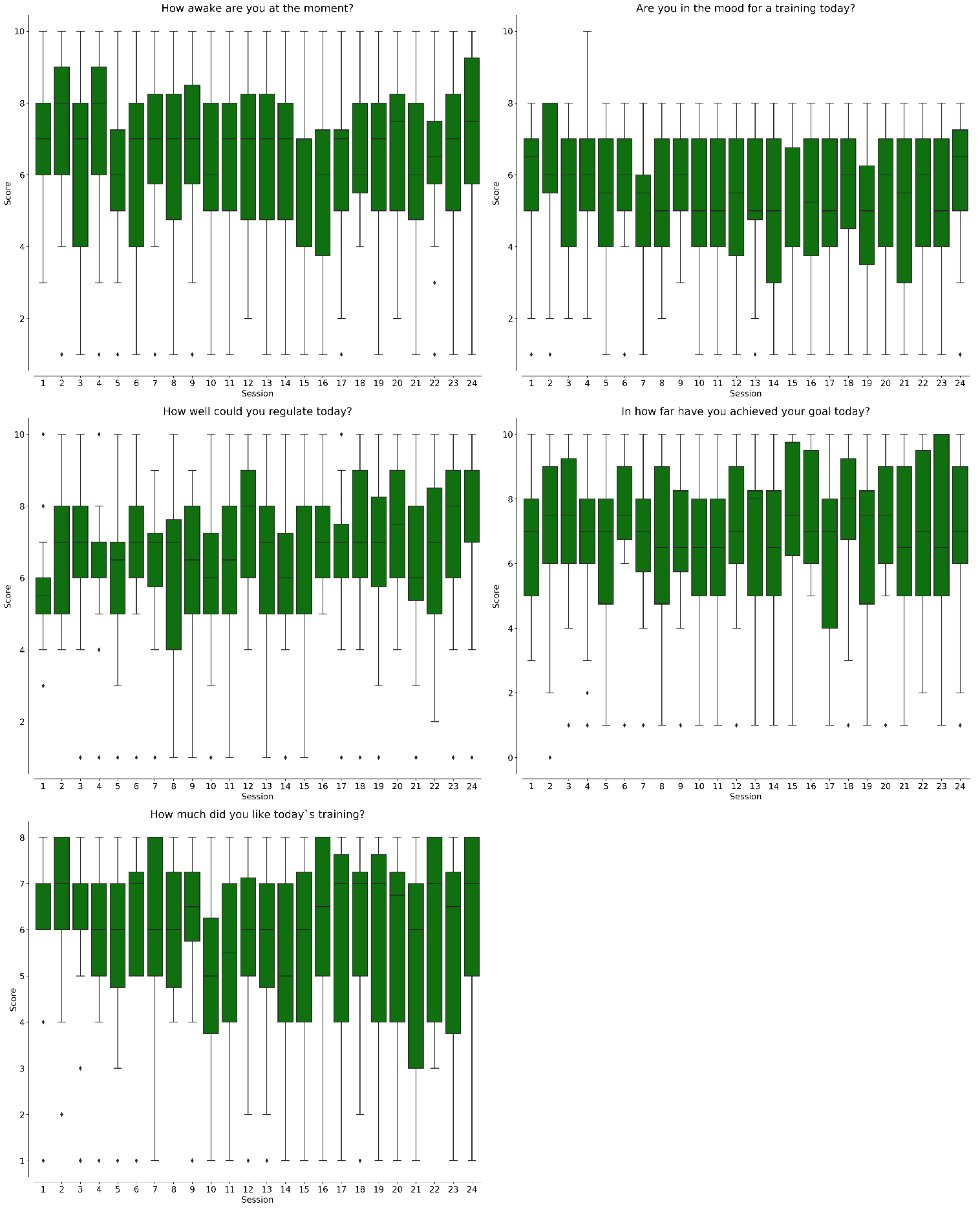


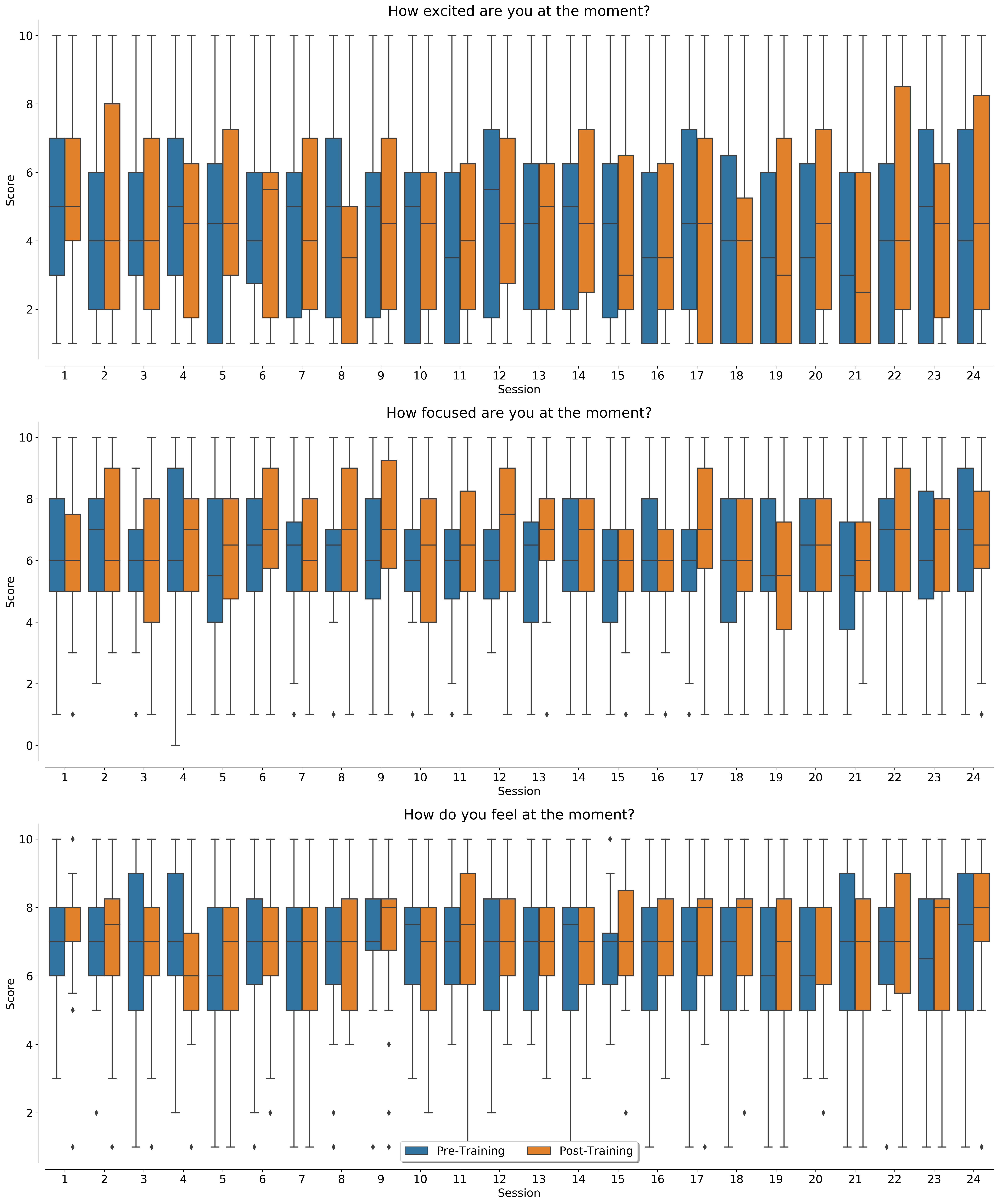


(E) Fragebogen zur Erfassung relevanter Therapiebedingungen (FERT) (Vollmann et al., 2009)

The assessment of diverse treatment-related trainer and participant variables suggests a constant stability of those nonspecific-effects:

*Experimental Condition*


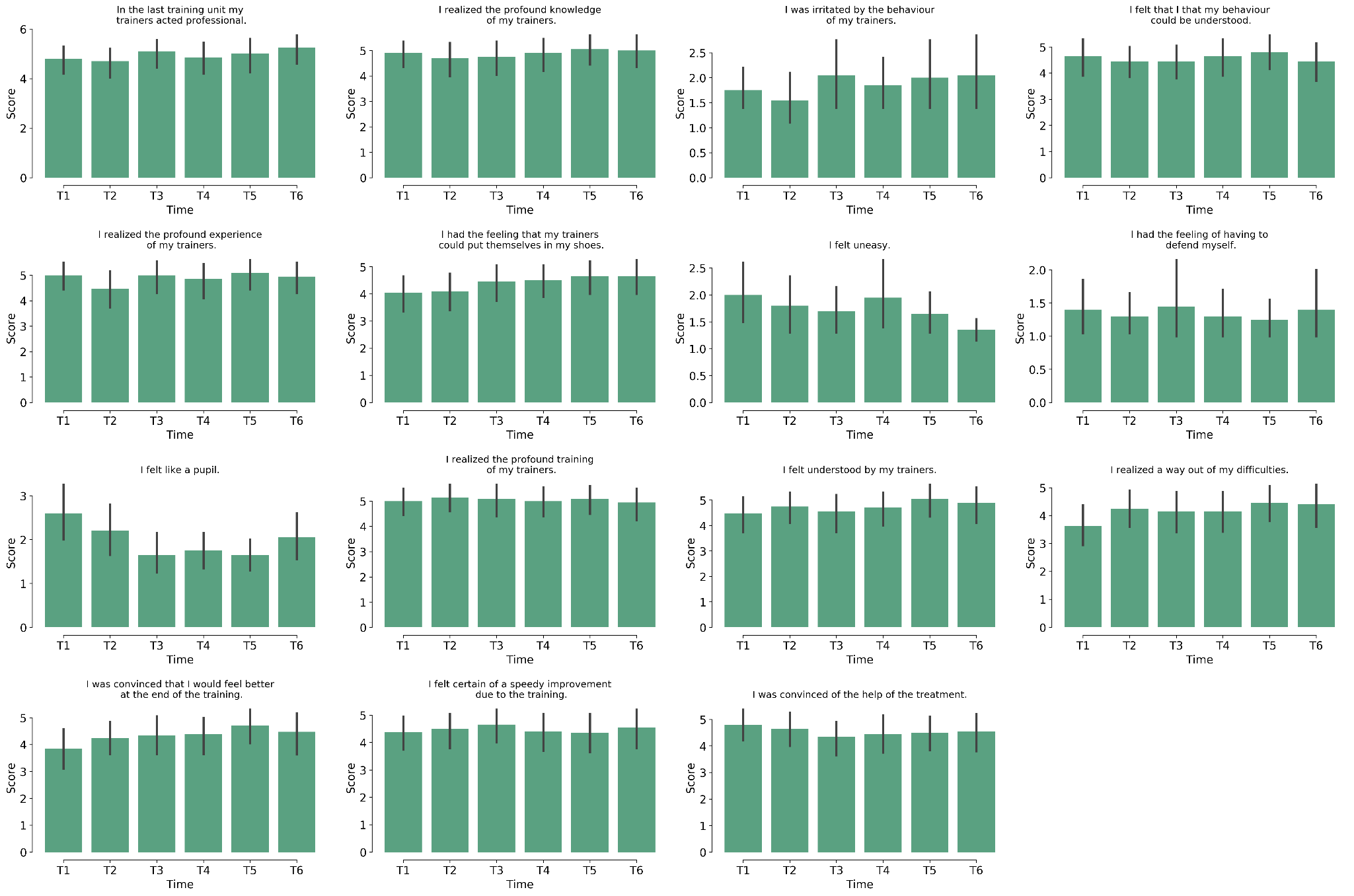


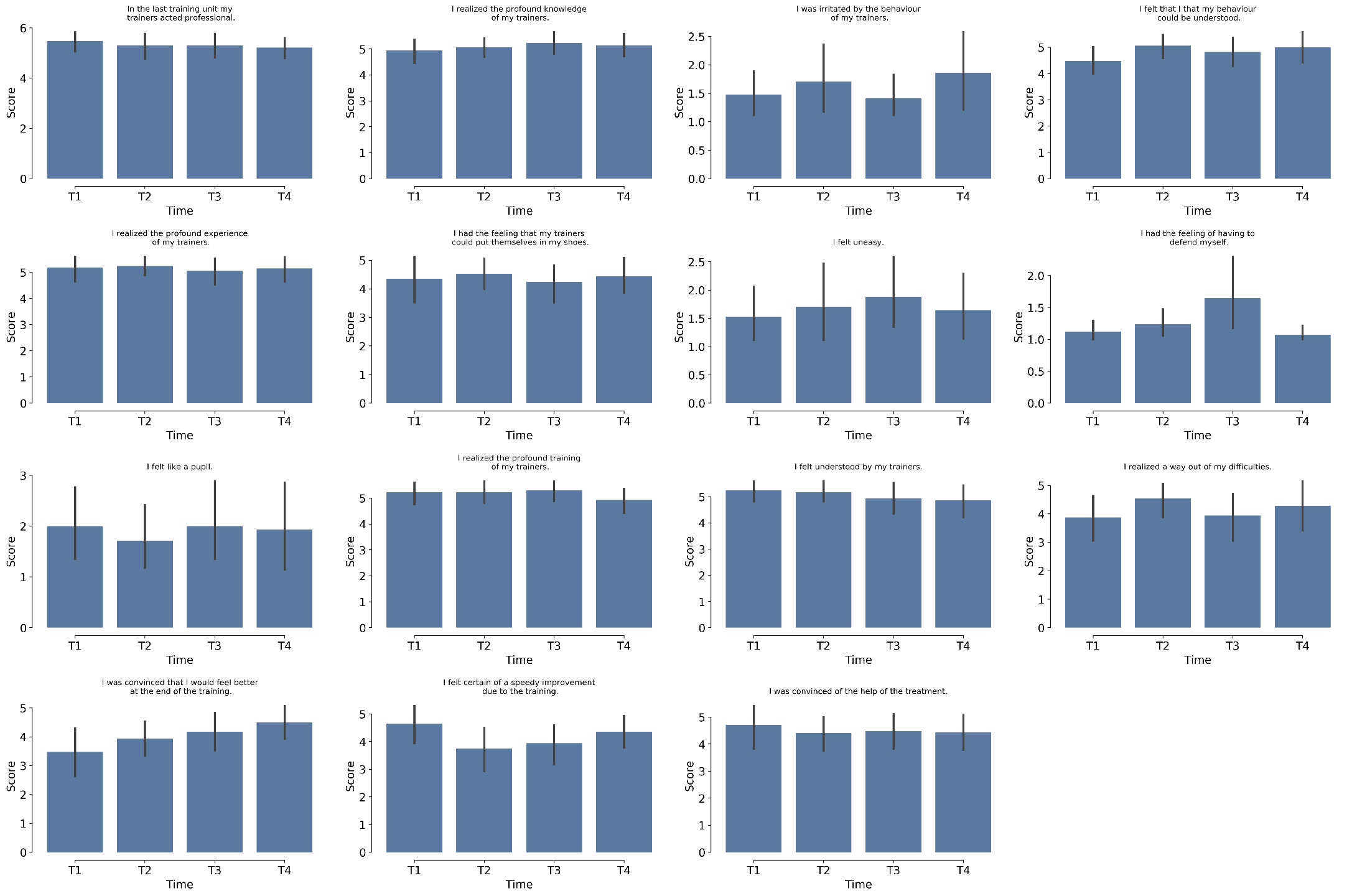
*Control Condition*

**(F) Detailed Multilevel Model Results**

**Table 1F.** Multilevel multivariate modeling results for the quadratic model of SRS subscale scores. Values in parenthesis indicate lower and upper bounds of the Bayesian 95% CI.

|  | **AM** | **SA** | **SCOM** | **SM** | **SCOG** |
| --- | --- | --- | --- | --- | --- |
| *Predictor* | *Estimate (CI)* | *Estimate (CI)* | *Estimate (CI)* | *Estimate (CI)* | *Estimate (CI)* |
| Intercept | 17.87 (15.45 – 20.33) | 12.78 (11.38 – 14.15) | 18.60 (16.46 – 20.69) | 35.01 (31.46 – 38.70) | 15.87 (13.78 – 17.95) |
| Time | -11.67 (-17.15 – -6.23) | -6.38 (-9.49 – -3.30) | -7.94 (-12.78 – -3.06) | -21.76 (-29.58 – -14.00) | -9.08 (-13.92 – -4.29) |
| Time^2^ | 7.85 (2.79 – 13.05) | 3.80 (0.89 – 6.76) | 3.60 (-0.99 – 8.12) | 14.58 (7.11 – 22.02) | 5.26 (0.64 – 9.83) |
| Group | -2.06 (-5.60 – 1.46) | -1.52 (-3.49 – 0.46) | -3.19 (-6.26 – -0.06) | -4.17 (-9.35 – 0.91) | 0.64 (-2.36 – 3.62) |
| Time x Group | 3.86 (-4.02 – 11.75) | 2.56 (-1.93 – 7.09) | -2.12 (-9.02 – 4.74) | 4.30 (-6.60 – 15.35) | 0.28 (-6.48 – 7.13) |
| Time^2^ x Group | -1.84 (-9.21 – 5.66) | -1.87 (-6.19 – 2.43) | 4.00 (-2.44 – 10.54) | -2.16 (-12.90 – 8.17) | 1.23 (-5.31 – 7.67) |
| **Random Effects** | | | | | |
| σ^2^ | 14.29 | | | | |
| τ_00_ | 6.05 | | | | |
| ICC | 0.71 | | | | |
| *N* | 41 | | | | |

*Note: σ^2^ denotes the within-person variability, τ_00_ denotes the between-person variability, and the ICC denotes the intra-class correlation, representing the proportion of variance in the outcome due to between-person differences.*

**Table 2F.** Multilevel multivariate modeling results for the linear model of SRS subscale scores. Values in parenthesis indicate lower and upper bounds of the Bayesian 95% CI.

|  | **AM** | **SA** | **SCOM** | **SM** | **SCOG** |
| --- | --- | --- | --- | --- | --- |
| *Predictor* | *Estimate (CI)* | *Estimates* | *Estimates* | *Estimates* | *Estimates* |
| Intercept | 16.80 (14.39 – 19.15) | 12.26 (10.89 – 13.59) | 18.11 (16.01 – 20.26) | 33.08 (29.51 – 36.68) | 15.19 (13.18 – 17.22) |
| Time | -3.78 (-5.73 – -1.84) | -2.57 (-3.56 – -1.59) | -4.36 (-6.00 – -2.71) | -7.18 (-9.92 – -4.51) | -3.83 (-5.42 – -2.29) |
| Group | -1.79 (-5.24 – 1.69) | -1.28 (-3.21 – 0.71) | -3.74 (-6.83 – -0.69) | -3.90 (-9.04 – 1.18) | 0.44 (-2.47 – 3.27) |
| Time x Group | 2.03 (-0.79 – 4.79) | 0.69 (-0.72 – 2.09) | 1.90 (-0.49 – 4.23) | 2.12 (-1.69 – 6.00) | 1.53 (-0.69 – 3.75) |
| **Random Effects** | | | | | |
| σ^2^ | 14.01 | | | | |
| τ_00_ | 6.60 | | | | |
| ICC | 0.69 | | | | |
| *N* | 41 | | | | |

*Note: σ^2^ denotes the within-person variability, τ_00_ denotes the between-person variability, and the ICC denotes the intra-class correlation, representing the proportion of variance in the outcome due to between-person differences.*

**Table 3F.** Multilevel modeling results for the linear and quadratic models of SRS total score.

|  | **Linear** | | **Quadratic** | |
| --- | --- | --- | --- | --- |
| *Predictor* | *Estimate* | *CI (95%)* | *Estimate* | *CI (95%)* |
| Intercept | 95.57 | 84.75 – 106.61 | 100.25 | 88.91 – 111.61 |
| Time | -21.59 | -31.61 – -11.62 | -56.49 | -77.00 – -34.74 |
| Group | -10.27 | -26.06 – 5.31 | -10.17 | -26.56 – 5.55 |
| Time x Group | 8.03 | -6.48 – 22.29 | 8.00 | -22.29 – 37.45 |
| Time^2^ |  |  | 35.02 | 17.55 – 51.87 |
| Time^2^ x Group |  |  | -0.27 | -24.20 – 24.48 |
| **Random Effects** | | | | |
| σ^2^ | 512.19 | | 525.69 | |
| τ_00_ | 166.22 | | 153.32 | |
| ICC | 0.76 | | 0.77 | |
| *N* | 41 | | 41 | |
| Marginal R^2^ / Conditional R^2^ | 0.083 / 0.847 | | 0.100 / 0.882 | |

*Note: σ^2^ denotes the within-person variability, τ_00_ denotes the between-person variability, and the ICC denotes the intra-class correlation, representing the proportion of variance in the outcome due to between-person differences.*

**Table 4F.** Multilevel modeling results for the linear and quadratic models of SCP learning curves.

|  | **Linear** | | **Quadratic** | |
| --- | --- | --- | --- | --- |
| *Predictors* | *Estimates* | *CI (95%)* | *Estimates* | *CI (95%)* |
| Intercept | -2.77 | -5.24 – -0.38 | -2.37 | -5.37 – 0.62 |
| Session | -0.62 | -4.25 – 3.00 | -3.27 | -14.72 – 8.53 |
| Positivity | 0.89 | -2.13 – 3.90 | 2.28 | -1.54 – 6.15 |
| Feedback 2 | -2.41 | -4.86 – 0.05 | -3.77 | -7.22 – -0.32 |
| Transfer | -1.82 | -4.24 – 0.59 | -2.24 | -5.74 – 1.20 |
| Session x Positivity | 3.25 | -1.06 – 7.59 | -5.31 | -21.26 – 10.51 |
| Session x Feedback 2 | 2.79 | -1.41 – 6.95 | 11.34 | -4.51 – 26.93 |
| Session x Transfer | 3.11 | -1.00 – 7.21 | 6.06 | -9.69 – 21.59 |
| Positivity x Feedback 2 | 1.03 | -2.34 – 4.48 | 1.81 | -3.01 – 6.76 |
| Positivity x Transfer | 2.35 | -1.08 – 5.78 | 1.47 | -3.33 – 6.40 |
| Session x Positivity x Feedback 2 | -3.59 | -9.49 – 2.46 | -8.54 | -31.34 – 13.68 |
| Session x Positivity x Transfer | -3.01 | -9.07 – 2.90 | 1.85 | -20.15 – 24.42 |
| Session^2^ |  |  | 2.69 | -8.73 – 13.67 |
| Session^2^ x Positivity |  |  | 8.61 | -6.70 – 23.93 |
| Session^2^ x Feedback 2 |  |  | -8.57 | -23.89 – 6.69 |
| Session^2^ x Transfer |  |  | -3.00 | -18.05 – 12.22 |
| Session^2^ x Positivity x Feedback 2 |  |  | 5.05 | -16.60 – 27.20 |
| Session^2^ x Positivity x Transfer |  |  | -4.86 | -26.37 – 16.35 |
| **Random Effects** | | | | |
| σ^2^ | 98.36 | | 97.78 | |
| τ_00_ | 15.54 _ID_ | | 14.20 _ID_ | |
| τ_11_ | 21.42 _ID x.Session_ | | 44.51 _ID x Session_ | |
|  | 15.82 _ID.x Positivity_ | | 44.79 _ID x Session_^2^ | |
|  | 3.50 _ID x Session x Positivity_ | | 15.97 _ID x Positivity_ | |
|  |  | | 5.26 _ID x Session x Positivity_ | |
|  |  | | 5.95 _ID x Session_^2^ _x Positivity_ | |
| ICC | 0.16 | | 0.23 | |
| *N* | 21 | | 21 | |
| Marginal R^2^ / Conditional R^2^ | 0.027 / 0.138 | | 0.031 / 0.146 | |

*Note: σ^2^ denotes the within-person variability, τ_00_ denotes the between-person variability in intercepts, τ_11_ and related terms denotes the between-person variability in random linear and quadratic terms across tasks, and ICC denotes the intra-class correlation, representing the proportion of variance in the outcome due to between-person differences.*

**Table 5F.** Multilevel modeling results for the quadratic models of task-related power spectral density

in the three lower frequency bands *delta*, *theta*, and *alpha.*

|  | **PSD Delta** | **PSD Theta** | **PSD Alpha** |
| --- | --- | --- | --- |
| *Predictors* | *Estimates*  *CI (95%)* | *Estimates*  *CI (95%)* | *Estimates*  *CI (95%)* |
| Intercept | 163.50 (140.87 – 185.77) | 40.39 (32.28 – 48.25) | 22.82 (15.91 – 29.57) |
| Session | -75.55 (-117.25 – -33.88) | 5.74 (-6.34 – 17.70) | 5.69 (-4.03 – 15.23) |
| Session^2^ | 62.87 (25.15 – 101.73) | -5.01 (-15.26 – 5.29) | -1.17 (-9.98 – 8.19) |
| Positivity | 2.78 (-9.51 – 15.06) | 1.14 (-1.51 – 3.78) | -0.34 (-2.79 – 2.10) |
| Feedback 2 | -4.86 (-17.49 – 8.17) | 0.94 (-1.67 – 3.57) | 0.18 (-2.17 – 2.61) |
| Transfer | -5.20 (-17.59 – 7.19) | -0.29 (-2.96 – 2.34) | -0.52 (-2.91 – 1.85) |
| Session x Positivity | -16.25 (-67.89 – 35.83) | -2.50 (-14.73 – 10.13) | -2.41 (-13.66 – 8.84) |
| Session^2^ x Positivity | 2.25 (-48.64 – 52.00) | 1.46 (-10.92 – 13.51) | -1.36 (-12.31 – 9.68) |
| Session x Feedback 2 | 9.58 (-44.99 – 62.97) | -4.50 (-16.73 – 7.77) | 0.14 (-11.01 – 11.22) |
| Session x Transfer | 42.42 (-10.49 – 96.39) | 3.68 (-8.58 – 15.93) | 4.75 (-6.35 – 15.83) |
| Session^2^ x Feedback 2 | -13.56 (-65.45 – 38.45) | 2.56 (-9.42 – 14.62) | -0.08 (-10.73 – 10.71) |
| Session^2^ x Transfer | -39.24 (-91.25 – 11.77) | -5.46 (-17.45 – 6.47) | -4.90 (-15.72 – 5.76) |
| Positivity x Feedback 2 | -8.61 (-25.96 – 8.25) | -0.45 (-4.18 – 3.28) | -0.69 (-4.13 – 2.70) |
| Positivity x Transfer | -3.20 (-20.33 – 14.02) | -0.27 (-4.00 – 3.49) | 0.98 (-2.41 – 4.31) |
| Session x Positivity x Feedback 2 | 37.00 (-35.10 – 108.87) | -1.53 (-19.09 – 15.71) | 3.70 (-11.85 – 19.55) |
| Session x Positivity x Transfer | 10.88 (-61.22 – 80.99) | -2.47 (-20.23 – 14.70) | -5.16 (-20.60 – 10.67) |
| Session^2^ x Positivity x Feedback 2 | -21.25 (-90.85 – 48.88) | 2.33 (-14.61 – 19.23) | -3.84 (-19.01 – 11.26) |
| Session^2^ x Positivity x Transfer | -3.24 (-70.77 – 67.14) | 2.55 (-14.28 – 19.61) | 7.92 (-7.42 – 23.10) |
| **Random Effects** | | | |
| σ^2^ | 1663.68 | 59.77 | 46.61 |
| τ_00_ | 2228.76 _ID_ | 324.11 _ID_ | 225.78 _ID_ |
| τ_11_ | 1406.62 _ID x Session_ | 365.33 _ID x Session_ | 165.56 _ID x Session_ |
|  | 542.50 _ID x Session_^2^ | 184.34 _ID x Session_^2^ | 130.39 _ID x Session_^2^ |
|  | 8.09 _ID x Positivity_ | 0.55 _ID x Positivity_ | 1.35 _ID x Positivity_ |
|  | 38.21 _ID x Session_ _x Positivity_ | 3.54 _ID x Session_ _x Positivity_ | 10.09 _ID x Session_ _x Positivity_ |
|  | 52.80 _ID x Session_^2^ _x Positivity_ | 7.84 _ID x Session_^2^ _x Positivity_ | 20.40 _ID x Session_^2^ _x Positivity_ |
| ICC | 0.51 | 0.90 | 0.88 |
| *N* | 21 | 21 | 20 |
| Marginal R^2^ / Conditional R^2^ | 0.019 / 0.463 | 0.003 / 0.822 | 0.008 / 0.843 |

*Note: σ^2^ denotes the within-person variability, τ_00_ denotes the between-person variability in intercepts, τ_11_ and related terms denotes the between-person variability in random linear and quadratic terms across tasks, and ICC denotes the intra-class correlation, representing the proportion of variance in the outcome due to between-person differences.*

**(G) Relationships Between Psychological Symptomatology Data (SRS) and Brain Data (SCP)**

**Statistical Analysis**

In order to model relationships between changes in SCP learning curves and changes in SRS, we followed a multivariate approach. We specified a multivariate multilevel regression model with two outcomes namely SCP amplitude and SRS total score, and explicitly modeled the pairwise correlations between the subject-specific coefficients (intercepts, linear and quadratic terms). For instance, a positive correlation between random intercepts in the SCP model and random intercepts in the SRS model indicate that individuals with high amplitudes at the beginning of training tend to have higher SRS scores at the beginning of training and vice versa. A positive correlation between linear terms would indicate initial rate of change in SCP amplitudes is associated with initial rate of change in SRS scores over individuals. On the other hand, a positive correlation between quadratic terms indicates that the direction and steepness of SCP amplitude curves over time is associated with the direction and steepness in SRS curves over individuals. We report all pairwise posterior correlations of interest and note that these multivariate analyses were performed in a purely exploratory manner. Thus, caution is needed when interpreting these results, which are best to be considered *merely as suggestive of further research*.

**Results**

The investigation of the relationships between SRS (total score) and SCP neurofeedback results revealed moderate to small posterior correlations indicating associations between SRS and SCP changes (only regarding negativity tasks which demands volitional generation of negative SCP shifts) over the course of training. Based on the previously presented SCP training results uncovering the prominent function of Feedback 1, the following correlational results are presented only for this significant condition (see **Fig. 1G**).

First, we could observe a positive correlation between SCP intercepts and SRS intercepts (first row), indicating that higher SRS scores at the beginning of the training are linked to less brain activity in the negativity task at the beginning of the training. Further, negative correlations indicate that higher SRS scores at the beginning of the training (SRS intercept) are associated with higher initial rates of change in SCP Negativity (SCP linear term; second row); further, higher SRS scores at the beginning of training are also associated with higher acceleration in learning to produce negative SCP shifts (SCP quadratic terms; third row).

In addition, the analysis revealed higher initial rates of decrease in SRS score (SRS linear terms) to be related to more positive SCP amplitudes in negativity tasks at the beginning of the training (SCP intercept, seventh row). Finally, in the fifteenth row, the positive correlation shows that the acceleration and steepness of SCP Negativity curves over time are positively related to the acceleration and steepness of SRS decrease curves over time.


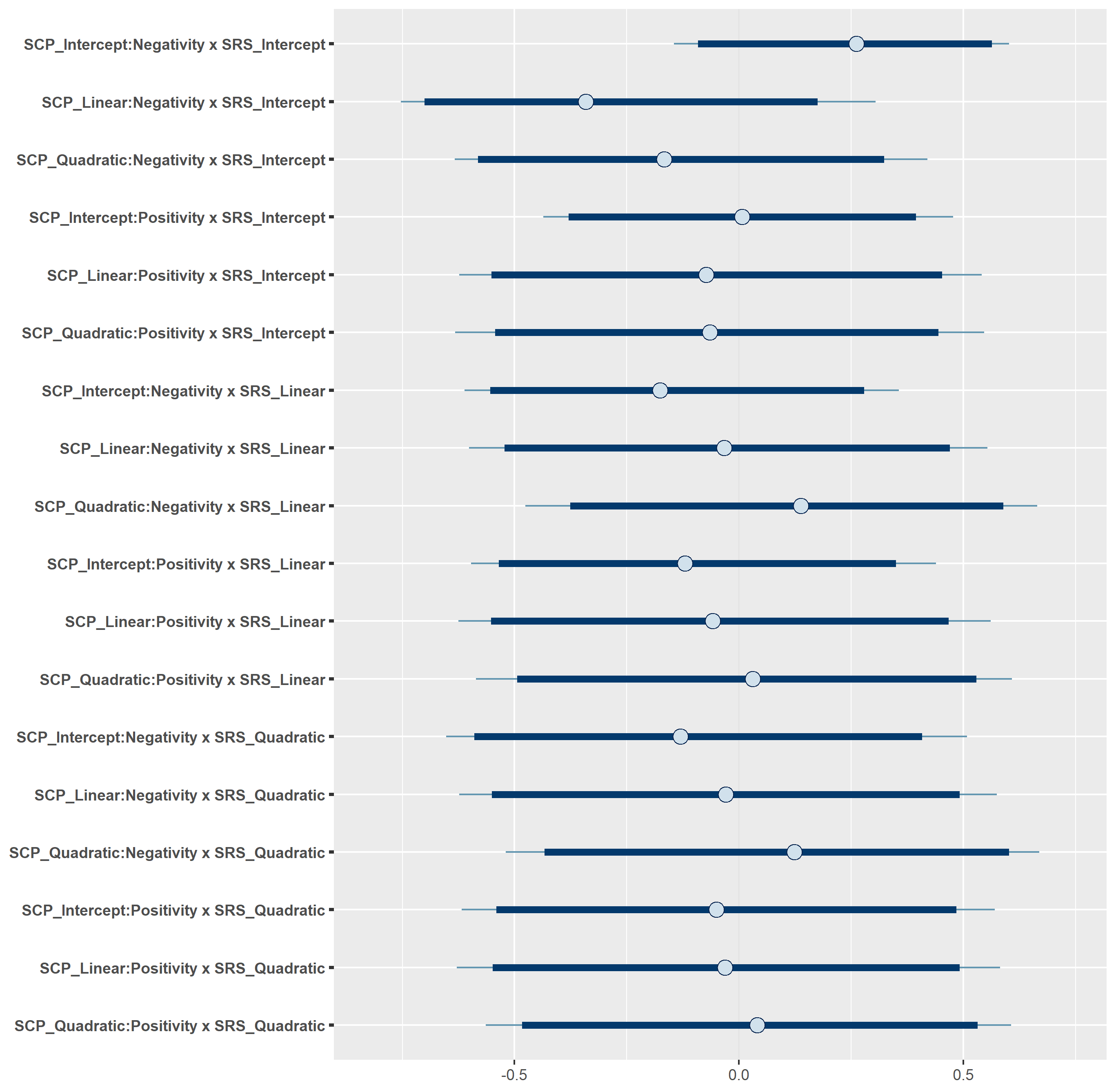


**Figure 1G. Correlational results from the multivariate model of psychological symptomatology and brain training data in the first feedback condition (SRS*SCP).** Each entry on the x-axis denotes a pair of model parameters from SCP and SRS models. The y-Axis depicts the estimated posterior correlations (the circles denote posterior medians; the thick blue lines represent a 95% CI; the light blue lines represent the range of the posterior).

In summary, the multivariate results of the analysis regarding relationship between autistic symptomatology and SCP neurofeedback training reveal a complex picture. It seems that adolescents with more severe ASD symptoms exhibit less cortical activation in the negativity task at the onset of training. This observation fits to the results of a general cortical hypoactivity (linked to a deficient production of negative SCP shifts and the reported attenuated CNV) in children and adolescents with ASD. Our analysis also shows that those participants scoring higher in SRS at the onset of training show better initial learning rates in terms of generating negative SCP shifts. Finally, SCP learning curves seem to be weakly associated with SRS change in terms of steepness and direction.

**(H) Posterior Predictive Checks**

**Figure 1H. Posterior predictive checks for the univariate multilevel model of SRS total score.** Global predictions are depicted. The black line indicates the global empirical distribution of the scale outcome. Shaded lines indicate model predictions using draws from the estimated posterior.


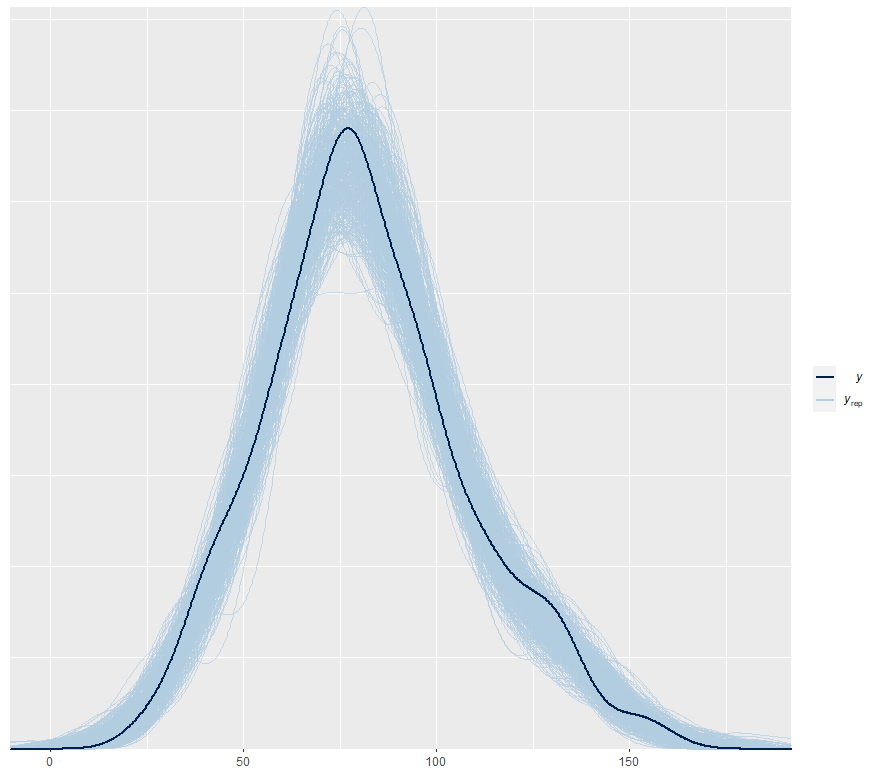


**Figure 2H. Posterior predictive checks for the univariate multilevel model of SRS total score.** Marginal individual predictions are depicted. The solid black lines indicate the empirical means of each participant. The shaded histograms depict individual posterior means computed using draws from the estimated posteriors.


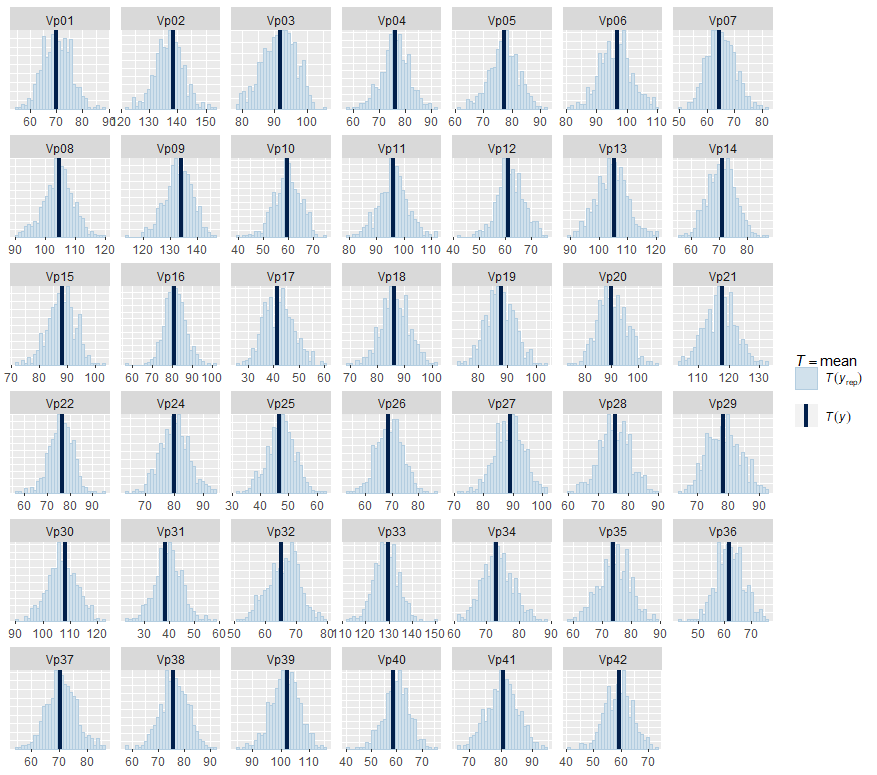


**Figure 3H. Posterior predictive checks for the univariate multilevel model of SRS total score**. Marginal individual predictions are depicted. The solid black lines indicate the empirical medians of each participant. The shaded histograms depict individual posterior medians computed using draws from the estimated posteriors.


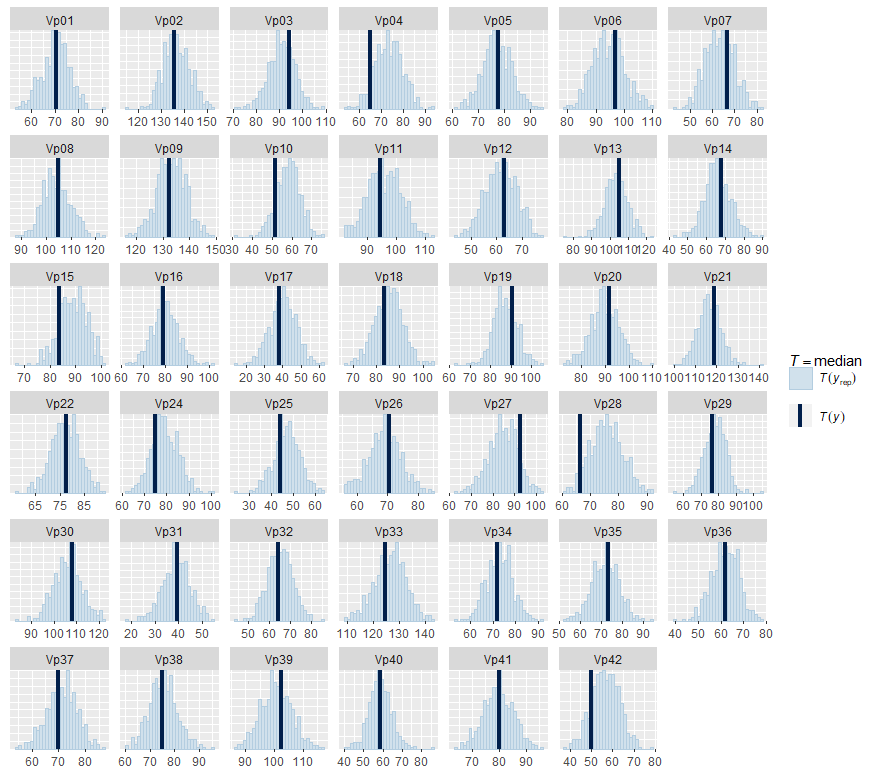


**Figure 4H. Posterior predictive checks for the multivariate multilevel model of SRS subscales.** Global predictions for subscale Social Cognition (SCOG) are depicted. The black line indicates the global empirical distribution of the scale outcome. Shaded lines indicate model predictions using draws from the estimated posterior.


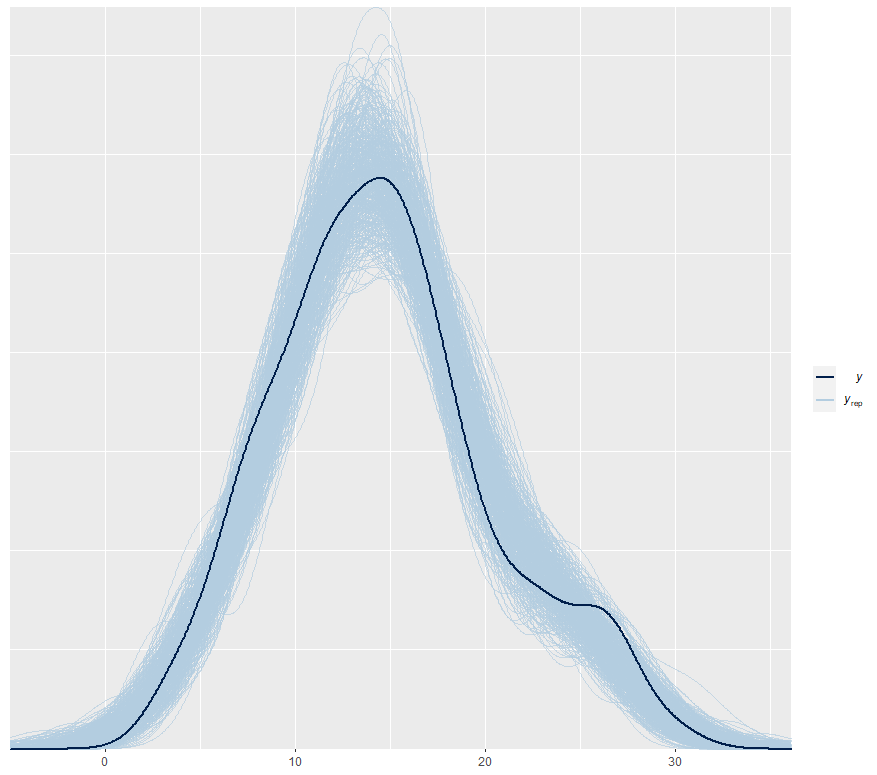


**Figure 5H. Posterior predictive checks for the multivariate multilevel model of SRS subscales.** Marginal individual predictions for the subscale Social Cognition (SCOG) are depicted. The solid black lines indicate the empirical means of each participant. The shaded histograms depict individual posterior means computed using draws from the estimated posteriors.


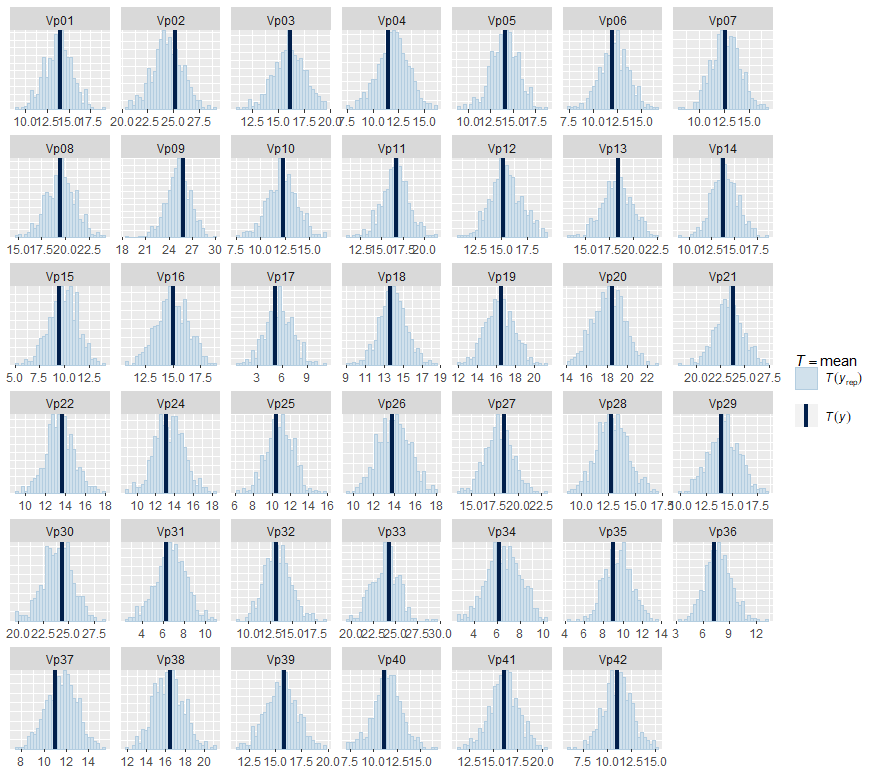


**Figure 6H. Posterior predictive checks for the multivariate multilevel model of SRS subscales**. Marginal individual predictions for the subscale Social Cognition (SCOG) are depicted. The solid black lines indicate the empirical medians of each participant. The shaded histograms depict individual posterior medians computed using draws from the estimated posteriors.


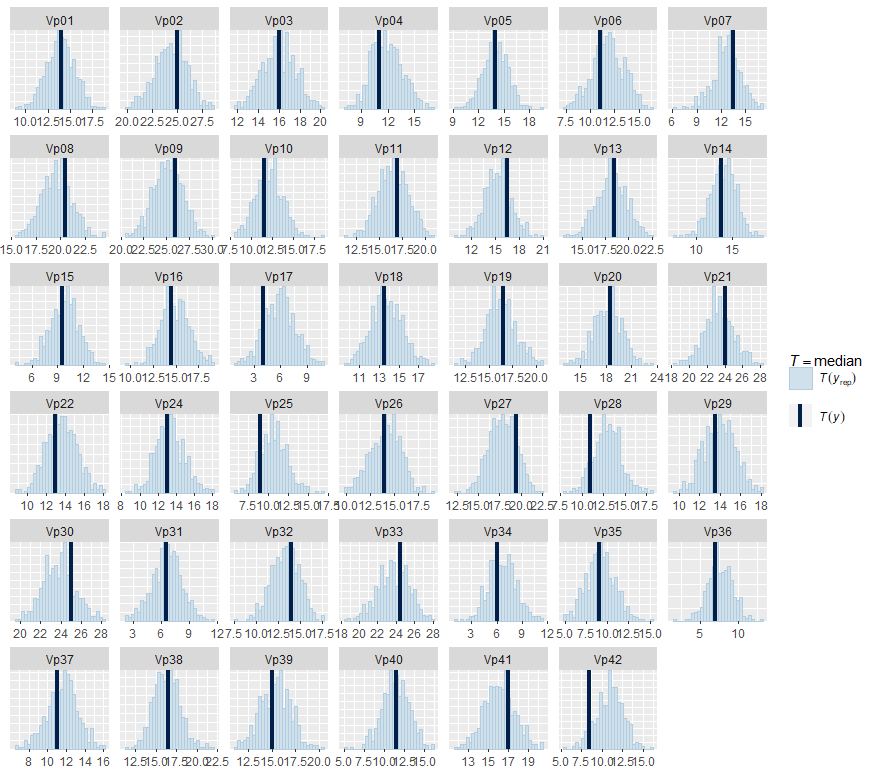


**Figure 7H. Posterior predictive checks for the multivariate multilevel model of SRS subscales.** Global predictions for subscale Autistic Mannerism (AM) are depicted. The black line indicates the global empirical distribution of the scale outcome. Shaded lines indicate model predictions using draws from the estimated posterior.


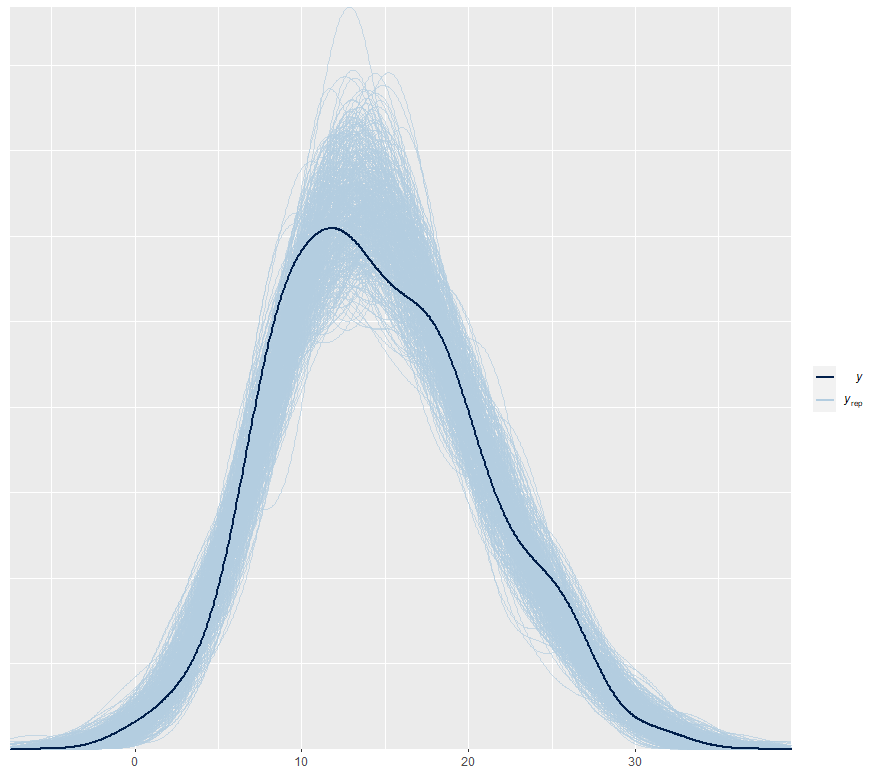


**Figure 8H. Posterior predictive checks for the multivariate multilevel model of SRS subscales.** Marginal individual predictions for the subscale Autistic Mannerism (AM) are depicted. The solid black lines indicate the empirical means of each participant. The shaded histograms depict individual posterior means computed using draws from the estimated posteriors.


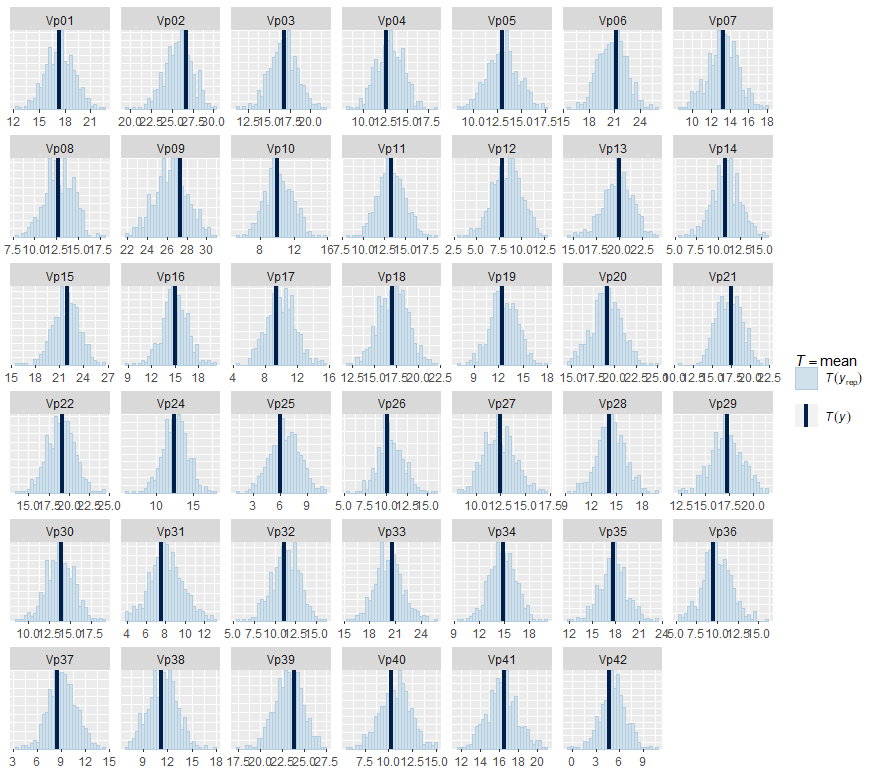


**Figure 9H. Posterior predictive checks for the multivariate multilevel model of SRS subscales**. Marginal individual predictions for the subscale Autistic Mannerism (AM) are depicted. The solid black lines indicate the empirical medians of each participant. The shaded histograms depict individual posterior medians computed using draws from the estimated posteriors.


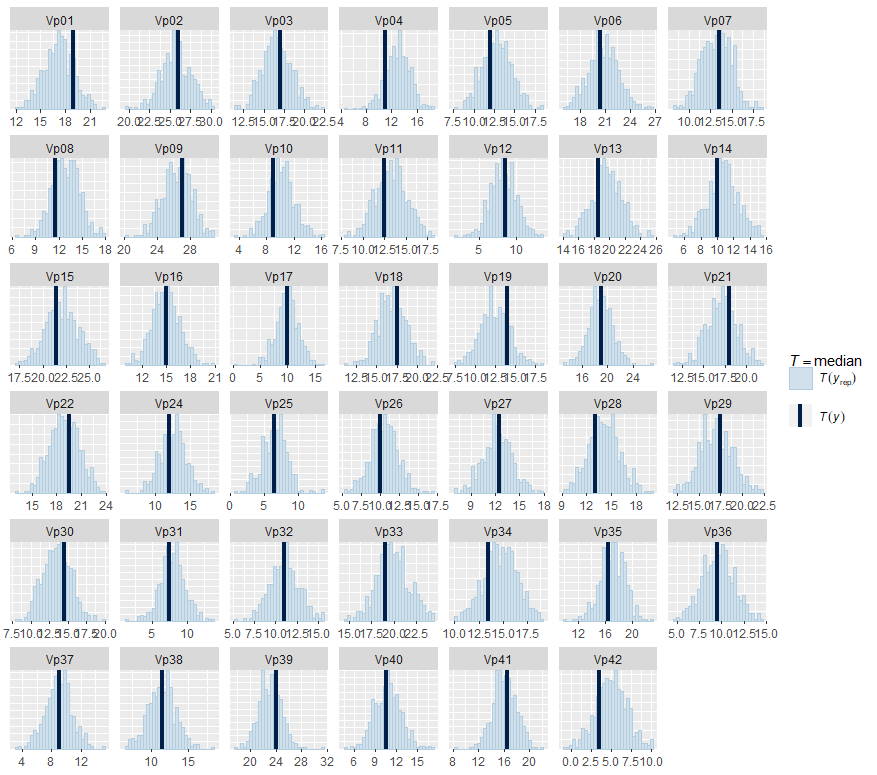


**Figure 10H. Posterior predictive checks for the multivariate multilevel model of SRS subscales.** Global predictions for subscale Social Motivation (SM) are depicted. The black line indicates the global empirical distribution of the scale outcome. Shaded lines indicate model predictions using draws from the estimated posterior.


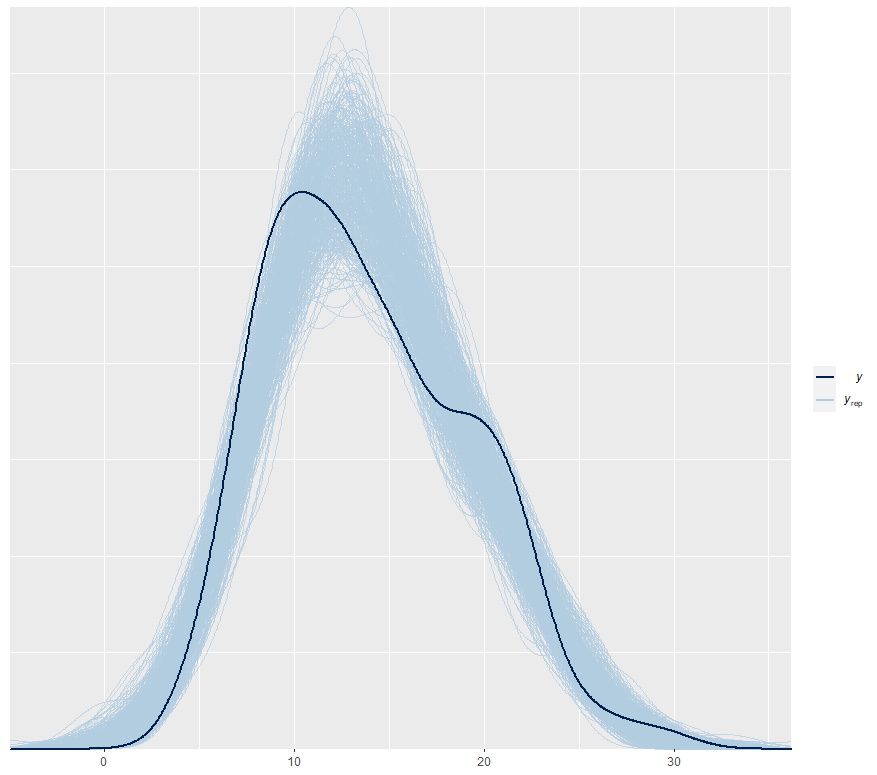


**Figure 11H. Posterior predictive checks for the multivariate multilevel model of SRS subscales.** Marginal individual predictions for the subscale Social Motivation (SM) are depicted. The solid black lines indicate the empirical means of each participant. The shaded histograms depict individual posterior means computed using draws from the estimated posteriors.


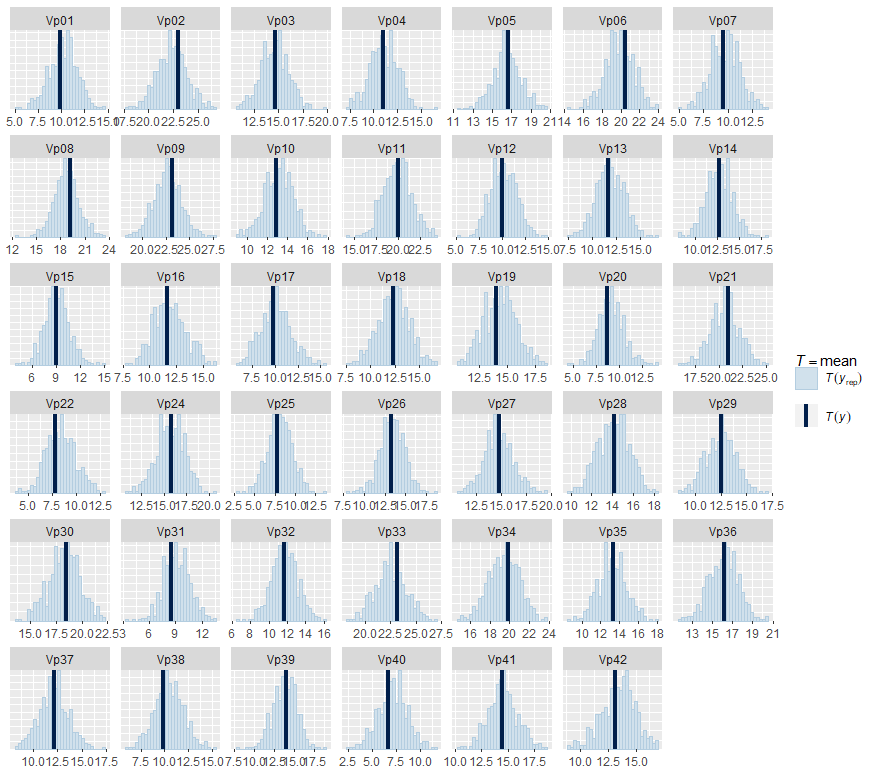


**Figure 12H. Posterior predictive checks for the multivariate multilevel model of SRS subscales**. Marginal individual predictions for the subscale Social Motivation (SM) are depicted. The solid black lines indicate the empirical medians of each participant. The shaded histograms depict individual posterior medians computed using draws from the estimated posteriors.


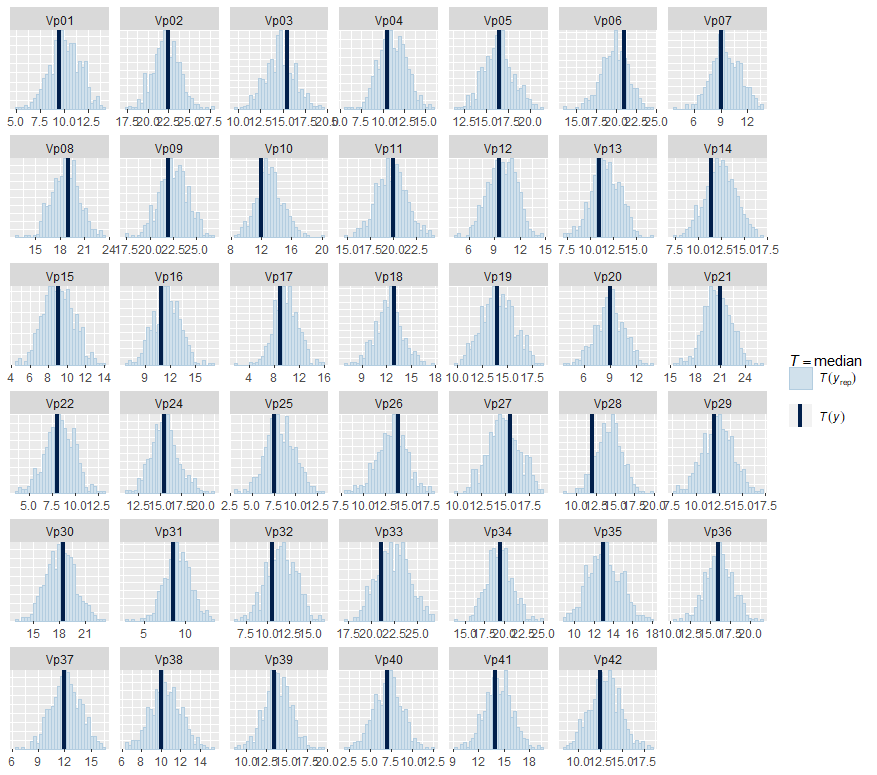


**Figure 13H. Posterior predictive checks for the multivariate multilevel model of SRS subscales.** Global predictions for subscale Social Communication (SCOM) are depicted. The black line indicates the global empirical distribution of the scale outcome. Shaded lines indicate model predictions using draws from the estimated posterior.


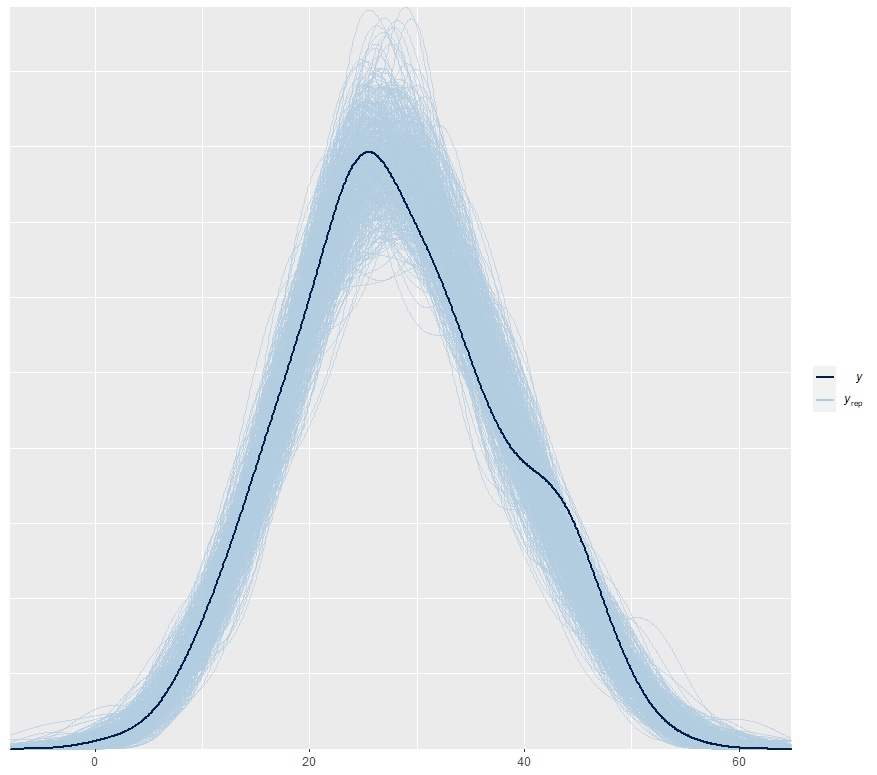


**Figure 14H. Posterior predictive checks for the multivariate multilevel model of SRS subscales**. Marginal individual predictions for the subscale Social Communication (SCOM) are depicted. The solid black lines indicate the empirical means of each participant. The shaded histograms depict individual posterior means computed using draws from the estimated posteriors.


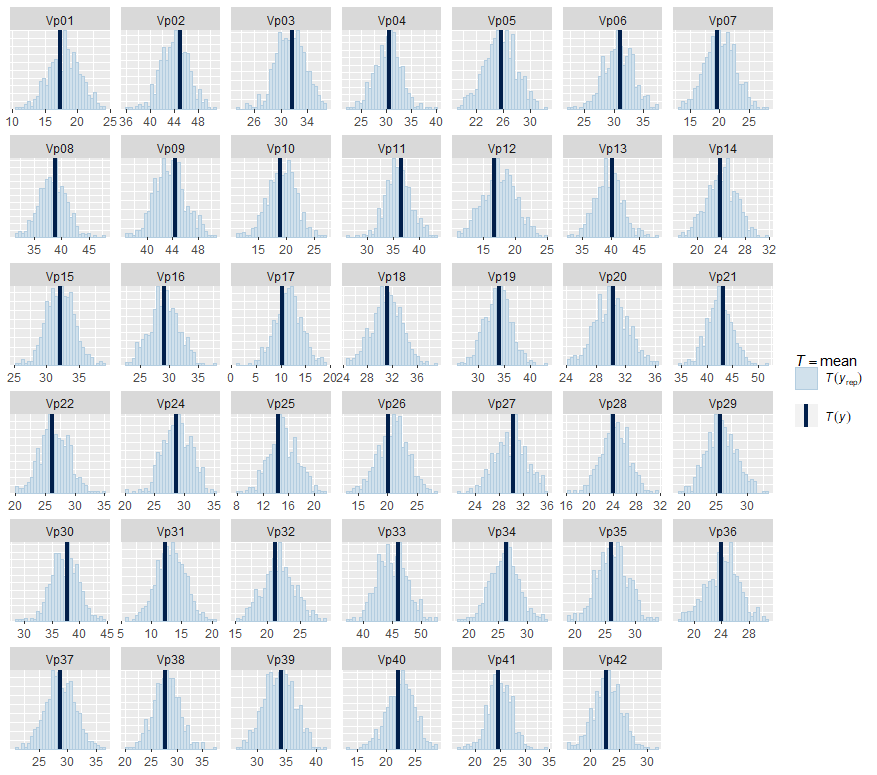


**Figure 15H. Posterior predictive checks for the multivariate multilevel model of SRS subscales**. Marginal individual predictions for the subscale Social Communication (SCOM) are depicted. The solid black lines indicate the empirical medians of each participant. The shaded histograms depict individual posterior medians computed using draws from the estimated posteriors.


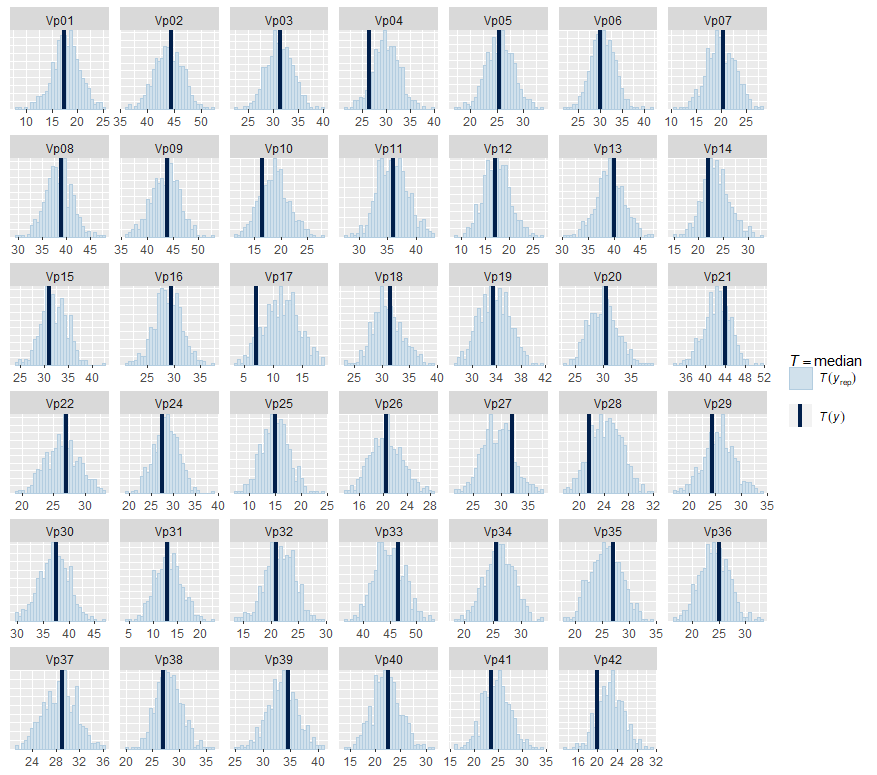


**Figure 16H. Posterior predictive checks for the multivariate multilevel model of SRS subscales.** Global predictions for subscale Social Awareness (SA) are depicted. The black line indicates the global empirical distribution of the scale outcome. Shaded lines indicate model predictions using draws from the estimated posterior.


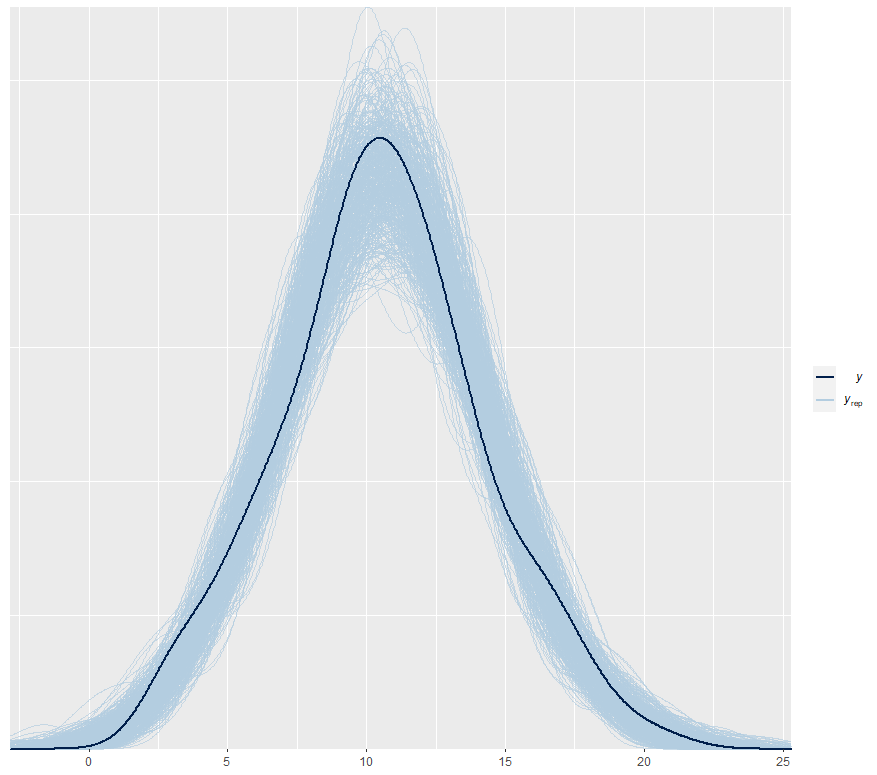


**Figure 17H. Posterior predictive checks for the multivariate multilevel model of SRS subscales**. Marginal individual predictions for the subscale Social Awareness (SA) are depicted. The solid black lines indicate the empirical means of each participant. The shaded histograms depict individual posterior means computed using draws from the estimated posteriors.


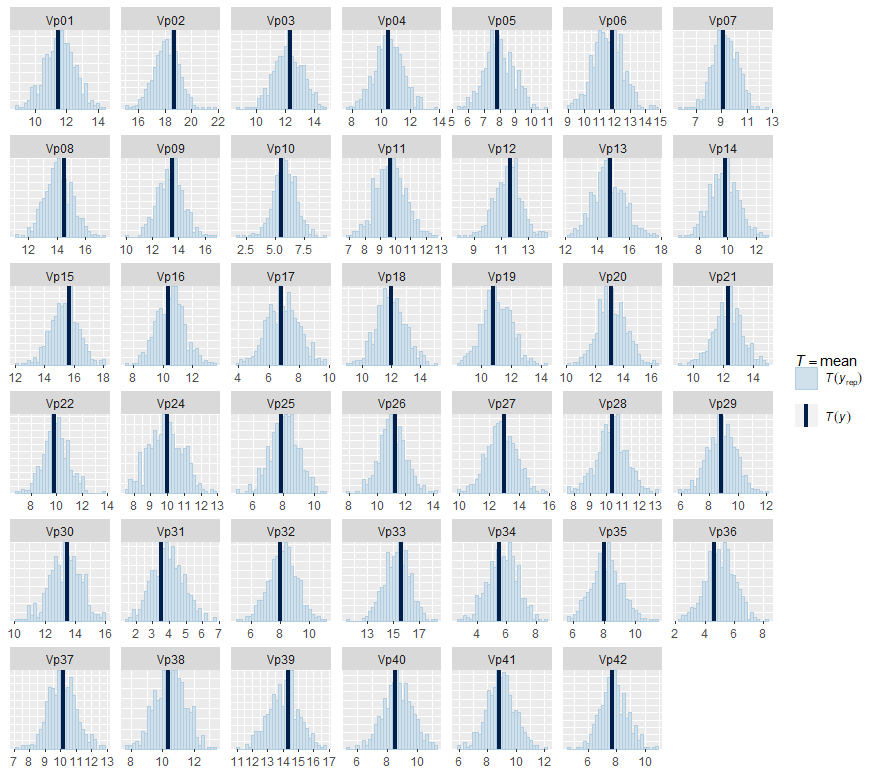


**Figure 18H. Posterior predictive checks for the multivariate multilevel model of SRS subscales**. Marginal individual predictions for the subscale Social Awareness (SA) are depicted. The solid black lines indicate the empirical medians of each participant. The shaded histograms depict individual posterior medians computed using draws from the estimated posteriors.


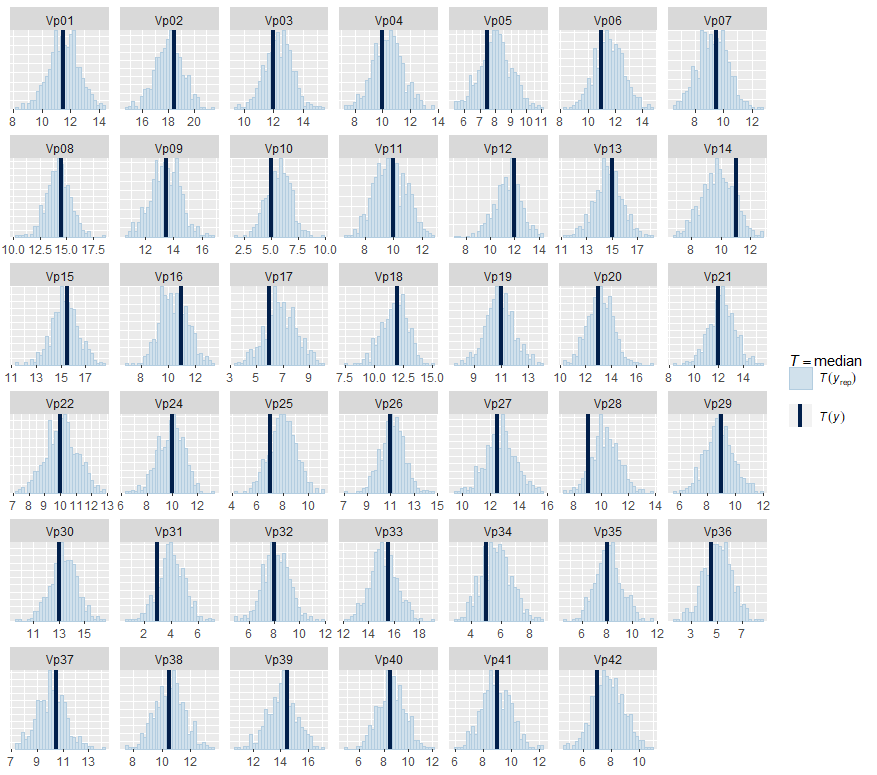


**Figure 19H. Posterior predictive checks for the multilevel model of SCP neurofeedback data.** Global predictions are depicted. The black line indicates the global empirical distribution of the scale outcome. Shaded lines indicate model predictions using draws from the estimated posterior.


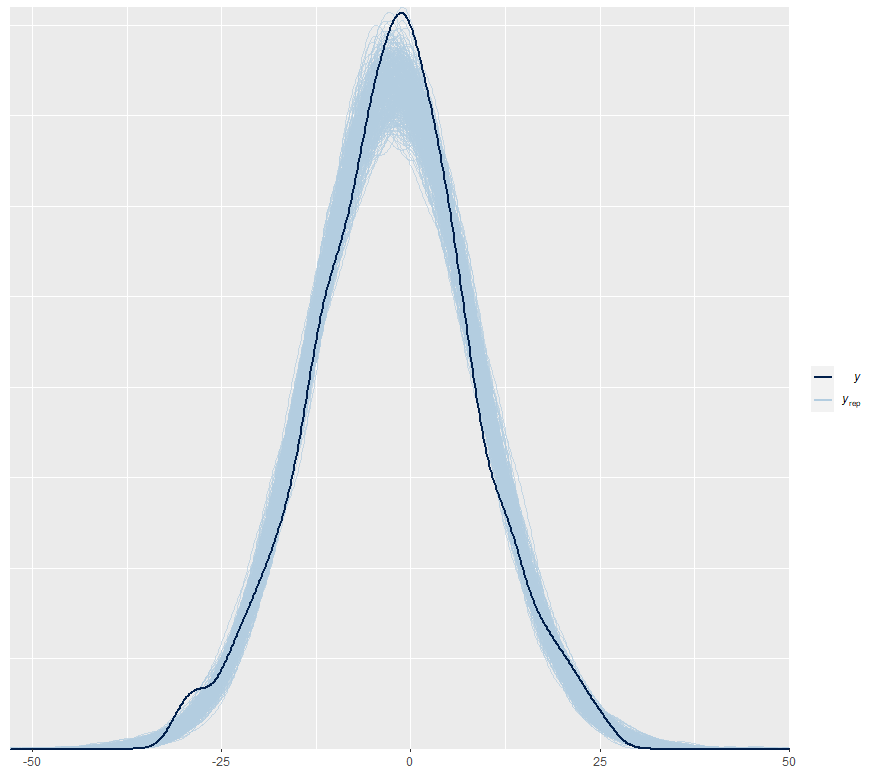


**Figure 20H. Posterior predictive checks for the multilevel model of SCP neurofeedback data.** Marginal individual predictions for mean SCP magnitude are depicted. The solid black lines indicate the empirical means of each participant. The shaded histograms depict individual posterior means computed using draws from the estimated posteriors.


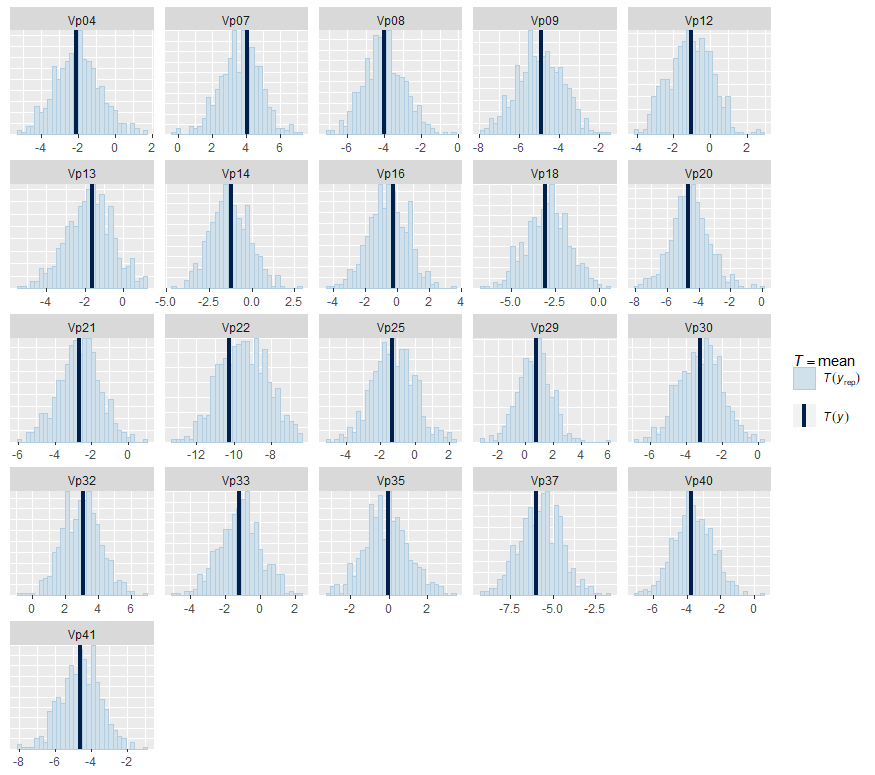


**Figure 21H. Posterior predictive checks for the multilevel model of SCP neurofeedback data.** Marginal individual predictions for median SCP magnitude are depicted. The solid black lines indicate the empirical medians of each participant. The shaded histograms depict individual posterior medians computed using draws from the estimated posteriors.


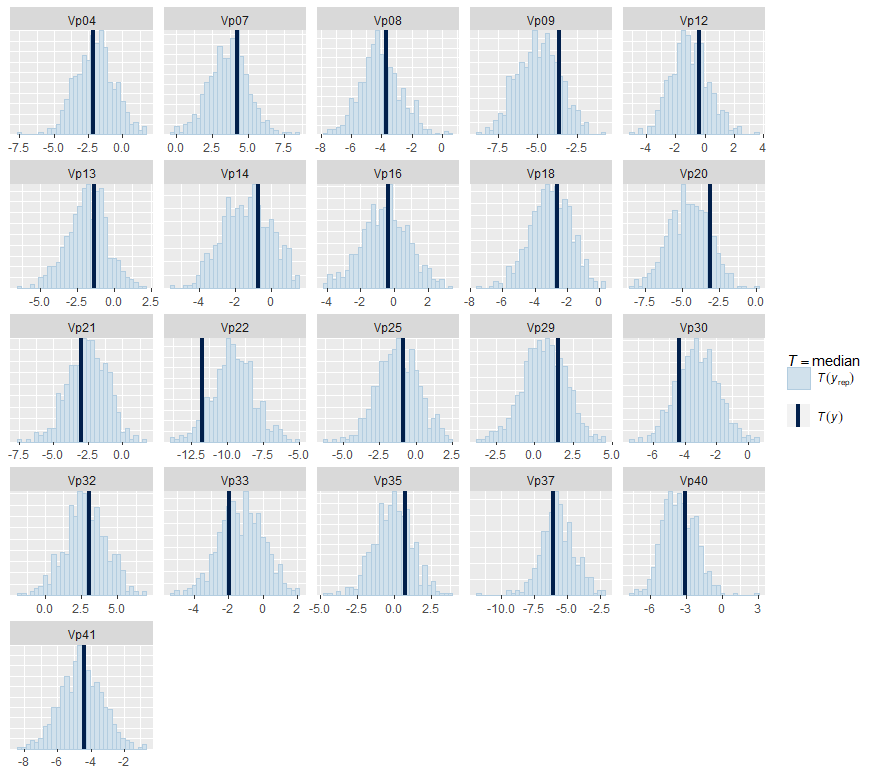


**Figure 22H. Posterior predictive checks for the multilevel model of task-related power spectral density in the delta frequency band.** Global predictions are depicted. The black line indicates the global empirical distribution of the scale outcome. Shaded lines indicate model predictions using draws from the estimated posterior.


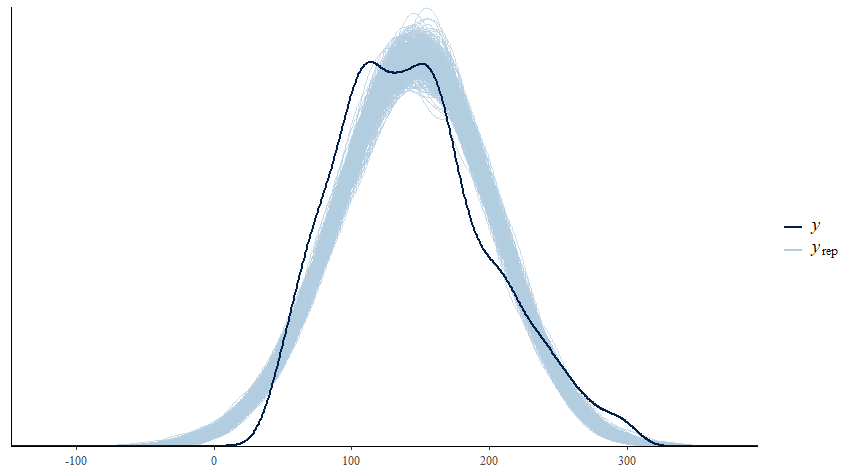


**Figure 23H. Posterior predictive checks for the multilevel model of task-related power spectral density in the delta frequency band.** Marginal individual predictions for mean PSD are depicted. The solid black lines indicate the empirical means of each participant. The shaded histograms depict individual posterior means computed using draws from the estimated posteriors.


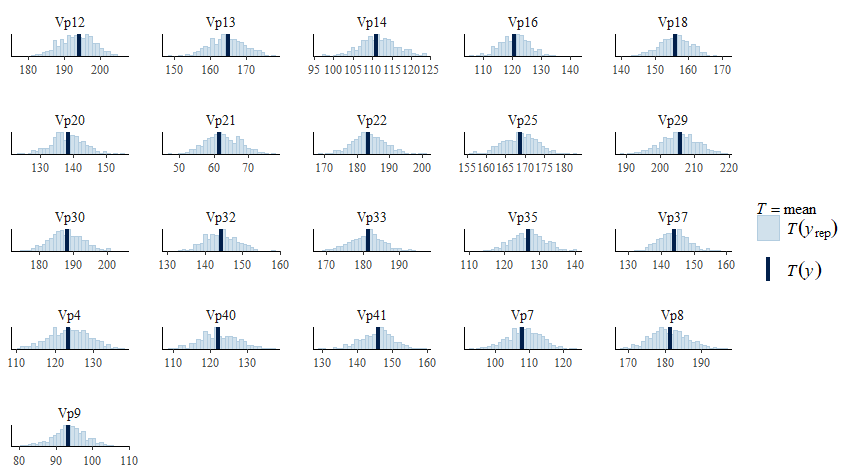


**Figure 24H. Posterior predictive checks for the multilevel model of task-related power spectral density in the delta frequency band**. Marginal individual predictions for median PSD are depicted. The solid black lines indicate the empirical medians of each participant. The shaded histograms depict individual posterior medians computed using draws from the estimated posteriors.


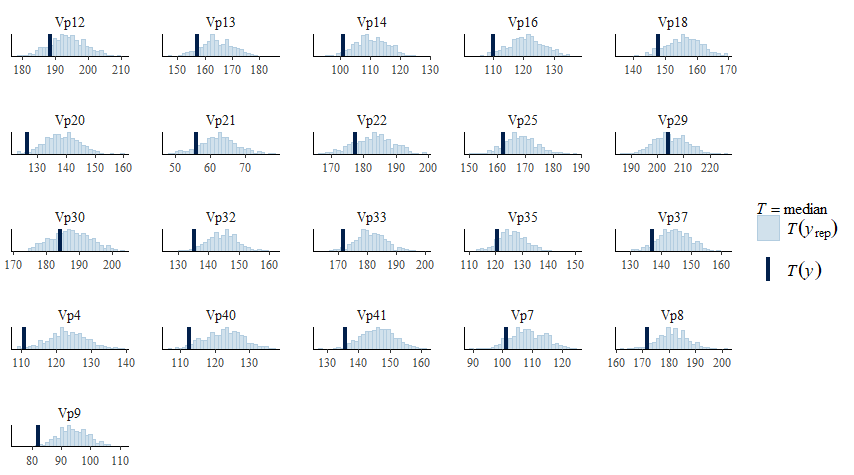


**Figure 22H. Posterior predictive checks for the multilevel model of task-related power spectral density in the delta frequency band.** Global predictions are depicted. The black line indicates the global empirical distribution of the scale outcome. Shaded lines indicate model predictions using draws from the estimated posterior.


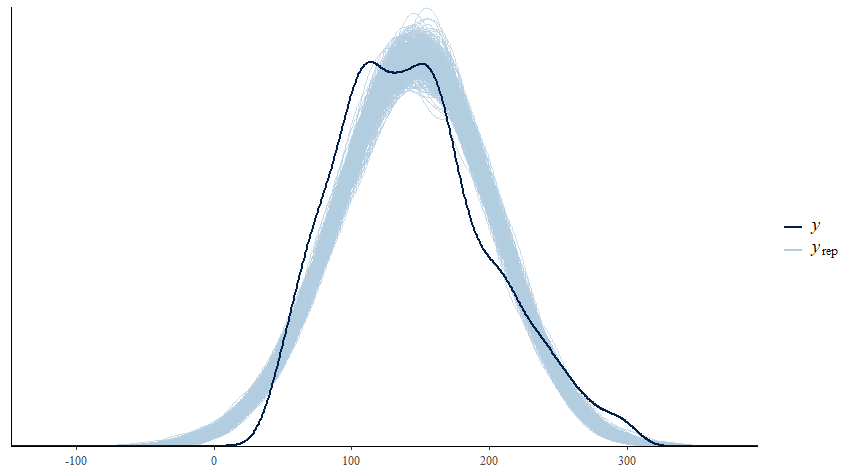


**Figure 23H. Posterior predictive checks for the multilevel model of task-related power spectral density in the delta frequency band.** Marginal individual predictions for mean PSD are depicted. The solid black lines indicate the empirical means of each participant. The shaded histograms depict individual posterior means computed using draws from the estimated posteriors.


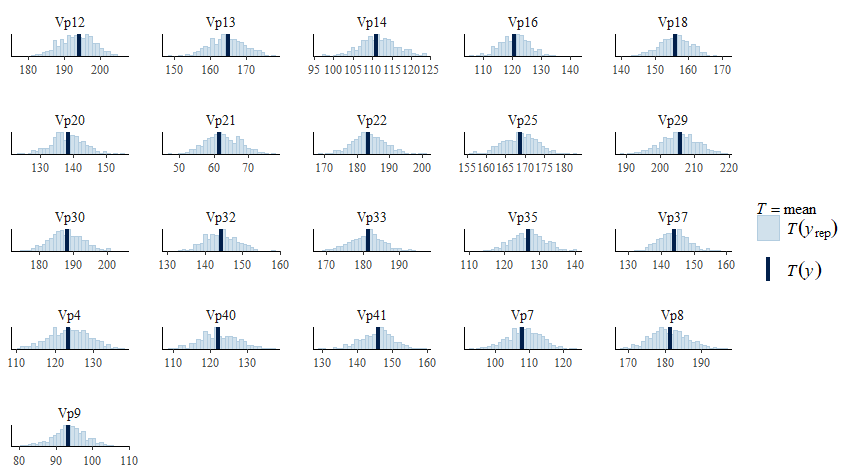


**Figure 24H. Posterior predictive checks for the multilevel model of task-related power spectral density in the delta frequency band**. Marginal individual predictions for median PSD are depicted. The solid black lines indicate the empirical medians of each participant. The shaded histograms depict individual posterior medians computed using draws from the estimated posteriors.


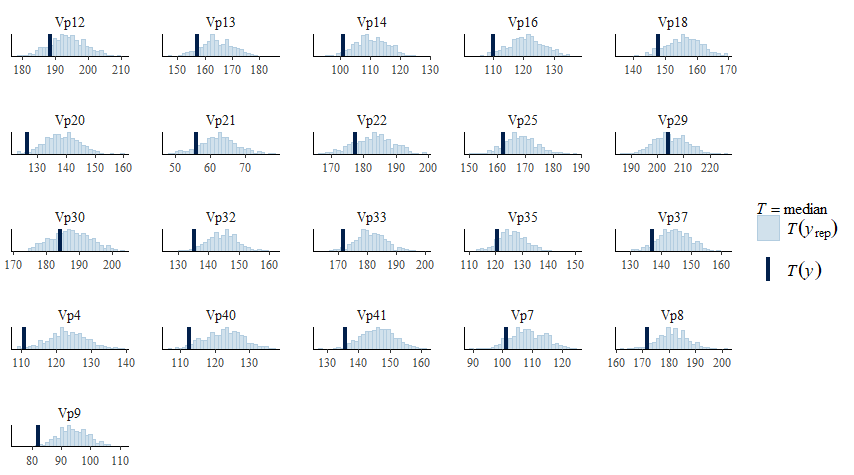


**Figure 25H. Posterior predictive checks for the multilevel model of task-related power spectral density in the theta frequency band.** Global predictions are depicted. The black line indicates the global empirical distribution of the scale outcome. Shaded lines indicate model predictions using draws from the estimated posterior.


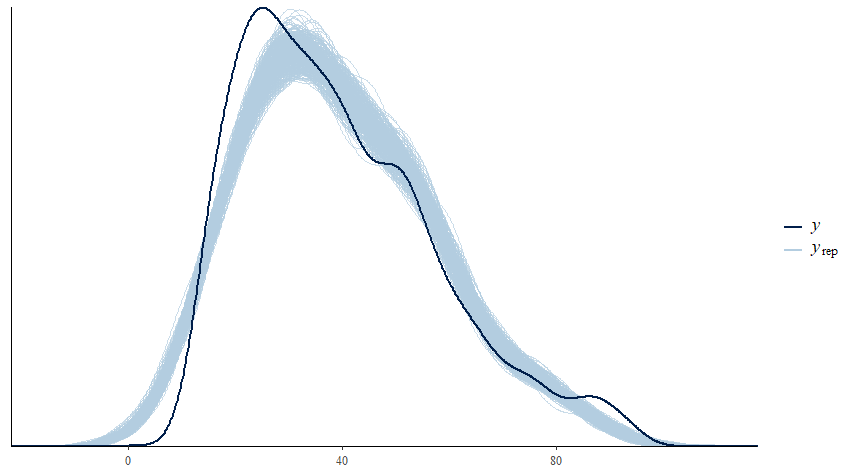


**Figure 26H. Posterior predictive checks for the multilevel model of task-related power spectral density in the theta frequency band.** Marginal individual predictions for mean PSD are depicted. The solid black lines indicate the empirical means of each participant. The shaded histograms depict individual posterior means computed using draws from the estimated posteriors.


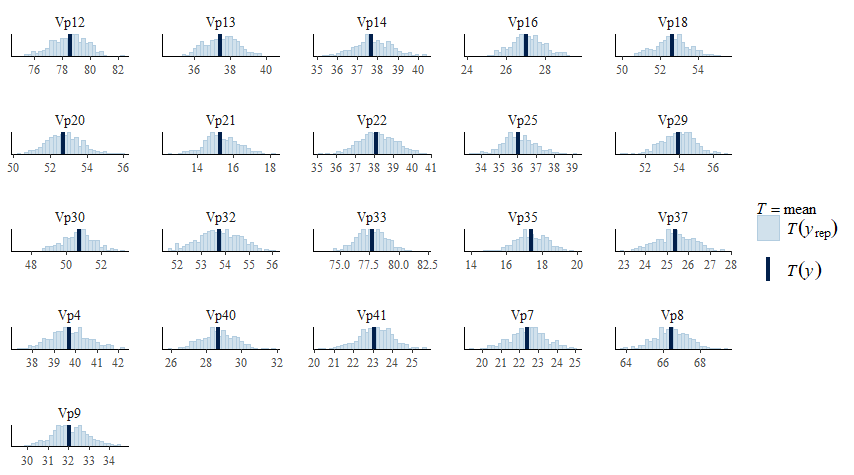


**Figure 27H. Posterior predictive checks for the multilevel model of task-related power spectral density in the theta frequency band**. Marginal individual predictions for median PSD are depicted. The solid black lines indicate the empirical medians of each participant. The shaded histograms depict individual posterior medians computed using draws from the estimated posteriors.


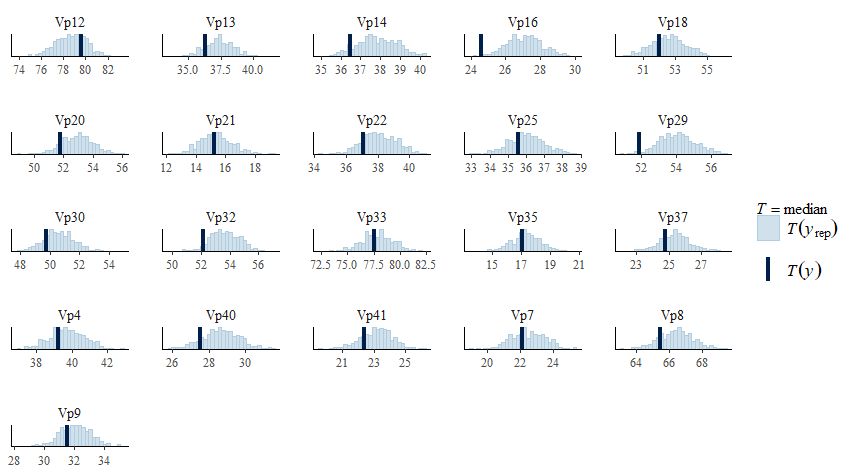


**Figure 28H. Posterior predictive checks for the multilevel model of task-related power spectral density in the alpha frequency band.** Global predictions are depicted. The black line indicates the global empirical distribution of the scale outcome. Shaded lines indicate model predictions using draws from the estimated posterior.


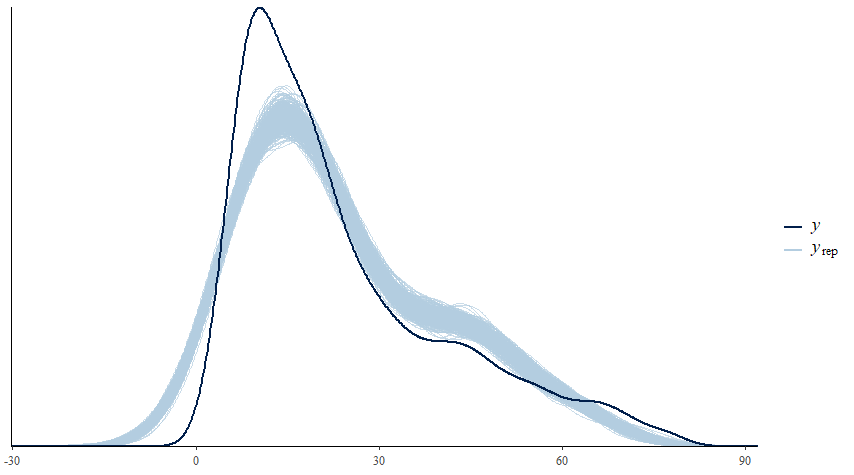


**Figure 29H. Posterior predictive checks for the multilevel model of task-related power spectral density in the alpha frequency band.** Marginal individual predictions for mean PSD are depicted. The solid black lines indicate the empirical means of each participant. The shaded histograms depict individual posterior means computed using draws from the estimated posteriors.


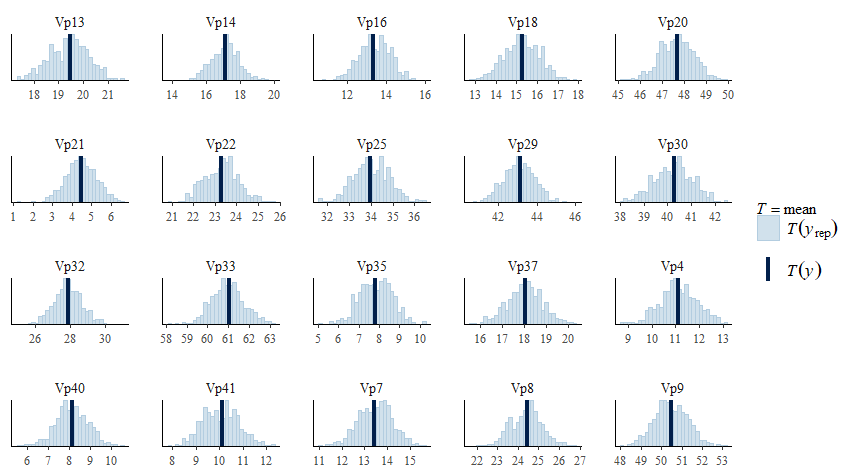


**Figure 30H. Posterior predictive checks for the multilevel model of task-related power spectral density in the alpha frequency band**. Marginal individual predictions for median PSD are depicted. The solid black lines indicate the empirical medians of each participant. The shaded histograms depict individual posterior medians computed using draws from the estimated posteriors.


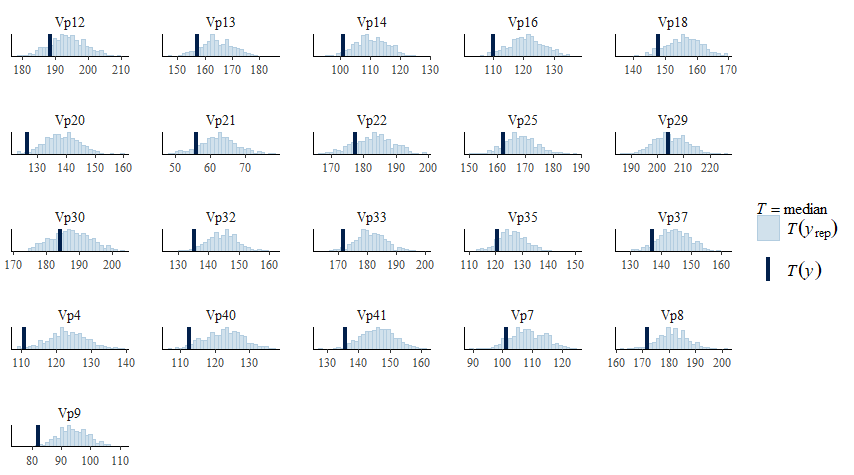

Supplement: Supplementary data 1 [file mmc1.docx]
